# Supplementary material for: DNA mini-barcoding reveals the mislabeling rate of canned cat food in Taiwan
Source: PeerJ. 2024 Feb 21;12:e16833. doi: 10.7717/peerj.16833 (PMC10893872; doi:10.7717/peerj.16833)
Supplement: Supplemental Information 1 [file peerj-12-16833-s001.pdf]

Table S1. List of all collected canned cat food products and results of BLAST analysis from *16S* rRNA barcodes.

Date	No.	Product code	Brand	Chinese label	English label	Declared ingredients (Chinese)	Place of manufacture	Tissue sample code	Sequencing method†	16S BLAST results	Mislabeled	Data access
2021.02.02	1	B1A	SEEDS	Hello Fresh 好鮮原汁湯罐 (清蒸鯖魚)	Mackerel	鯖魚	Thailand	B1A	Sanger (+)	*Decapterus russelli*	Yes	Supplementary Information 2
										Decapterus maruadsi		
										Caranx sexfasciatus		
										Selar crumenophthalmus		
										Trachurus novaezelandiae		
	2	B1B	SEEDS	Hello Fresh 好鮮原汁湯罐 (清蒸鮪魚)	Tuna	鮪魚	Thailand	B1B	Sanger (+)	*Katsuwonus pelamis*	No	Supplementary Information 2
										Thunnus albacares[a]		
	3	B1C	SEEDS	Tuna 愛貓天然食（兩倍鮮嫩雞肉＋白身鮪魚）	Chicken & Tuna light meat	白身鮪魚	Thailand	B1C1	Sanger (+)	*Katsuwonus pelamis*	No	Supplementary Information 2
						雞肉				*Thunnus albacares*[a]		
								B1C2	Sanger (+)	*Gallus gallus*		Supplementary Information 2

	4	B1D	YAMI 亞米	健寶 鮪魚蟹柳活力餐		鮪魚	Thailand	B1D1	Sanger (+)	*Euthynnus affinis*	Yes	Supplementary Information 2
						蟹柳		B1D2	Sanger (-)*			
2021.02.23	5	B2A	SEEDS	MamaMia 貓餐罐	Chicken	雞肉	Thailand	B2A	Sanger (+)	*Gallus gallus*	No	Supplementary Information 2
	6	B2B	SEEDS	MamaMia 貓餐罐	Chicken + tuna light meat + tomato	雞肉	Thailand	B2B1	Sanger (+)	*Gallus gallus*	Yes	Supplementary Information 2
						白身鮪魚		B2B2	Sanger (+)	*Euthynnus lineatus*		Supplementary Information 2
	7	B2F	SEEDS	Tuna 愛貓天然食	Light tuna meat & shirasu	白身鮪魚	Thailand	B2F1	Sanger (+)	*Katsuwonus pelamis*	No	Supplementary Information 2
						吻仔魚				*Thunnus albacares*[a]		
								B2F2	Sanger (+)	*Encrasicholina punctifer*		Supplementary Information 2
	8	B2G	SEEDS	Bistro Cat 特級銀貓健康餐罐	Tuna light meat + shrimp	白身鮪魚	Thailand	B2G1	Sanger (+)	*Katsuwonus pelamis*	No	Supplementary Information 2
						蝦肉				*Thunnus albacares*[a]		
								B2G2	Sanger (-)*			

	9	B2H	SEEDS	Hello Fresh 好鮮原汁湯罐(清蒸雞肉&牛肉)	Chicken & beef	雞肉	Thailand	B2H1	Sanger (+)	*Gallus gallus*	No	Supplementary Information 2
						牛肉		B2H2	Sanger (+)	*Bos taurus*		Supplementary Information 2
2021.02.26	10	B3A	GOEN	御宴湯罐 白身鮪魚+雞肉		白身鮪魚	Thailand	B3A1	Sanger (+)	*Katsuwonus pelamis*	No	Supplementary Information 2
						雞肉				*Thunnus albacares*[a]		
								B3A2	Sanger (+)	*Gallus gallus*		Supplementary Information 2
	11	B3B	GOEN	御宴湯罐 白身鮪魚+鮭魚		白身鮪魚	Thailand	B3B1	Sanger (+)	*Katsuwonus pelamis*	No	Supplementary Information 2
						鮭魚				*Thunnus albacares*[a]		
								B3B2	Sanger (+)	*Salmo salar*		Supplementary Information 2
	12	B3C	GOEN	御宴湯罐 白身鮪魚+鯛魚		白身鮪魚	Thailand	B3C1	Sanger (+)	*Katsuwonus pelamis*	No	Supplementary Information 2
						鯛魚				*Thunnus albacares*[a]		
								B3C2	Sanger (+)	*Priacanthus tayenus*		Supplementary Information 2

13	B3D	GOEN	御宴湯罐 白身鮪魚+鮮蝦	白身鮪魚	Thailand	B3D1	Sanger (+)	*Katsuwonus pelamis*	No	Supplementary Information 2
				鮮蝦				*Thunnus albacares*[a]		
						B3D2	Sanger (+)	*Metapenaeopsis barbata*		Supplementary Information 2
								Metapenaeopsis palmensis		
								Metapenaeopsis stridulans		
14	B3E	GOEN	御宴湯罐 白身鮪魚+吻仔魚	白身鮪魚	Thailand	B3E1	Sanger (+)	*Katsuwonus pelamis*	No	Supplementary Information 2
				吻仔魚				*Thunnus albacares*[a]		
						B3E2	Sanger (+)	*Engraulis japonicus*		Supplementary Information 2
15	B3F	元氣家族	元氣家族金罐 鮪魚+鯛魚	鮪魚	Thailand	B3F1	Sanger (+)	*Euthynnus affinis*	Yes	Supplementary Information 2
				鯛魚				*Euthynnus lineatus*[b]		
								Auxis thazard[c]		
						B3F2	Sanger (+)	*Priacanthus tayenus*		Supplementary Information 2
16	B3G	元氣家族	元氣家族金罐 鮪魚+吻仔魚	鮪魚	Thailand	B3G1	Sanger (-)*		No	

				吻仔魚		B3G2	Sanger (+)	*Engraulis japonicus*		Supplementary Information 2
17	B3H	元氣家族	元氣家族金罐 鮪魚+鮮蝦	鮪魚	Thailand	B3H1	Sanger (+)	*Euthynnus affinis*	Yes	Supplementary Information 2
				鮮蝦				*Euthynnus lineatus*[b]		
								Auxis thazard[c]		
						B3H2	Sanger (-)*			
18	B3I	元氣家族	元氣家族金罐 鮪魚+雞肉	鮪魚	Thailand	B3I1	Sanger (+)	*Euthynnus affinis*	Yes	Supplementary Information 2
				雞肉				*Euthynnus lineatus*[b]		
								Auxis thazard[c]		
						B3I2	Sanger (+)	*Gallus gallus*		Supplementary Information 2
19	B3J	元氣家族	元氣家族金罐 鮪魚+蟹肉棒	鮪魚	Thailand	B3J1	Sanger (+)	*Euthynnus affinis*	Yes	Supplementary Information 2
								Euthynnus lineatus[b]		
								Auxis thazard[c]		
				蟹肉棒		B3J2	Sanger (+)	*Euthynnus lineatus*		Supplementary Information 2
								Euthynnus lineatus[b]		

									Auxis thazard[c]		
	20	B3K	元氣家族	元氣家族金罐 鮪魚+鮭魚	鮪魚	Thailand	B3K1	Sanger (+)	*Euthynnus affinis*	Yes	Supplementary Information 2
					鮭魚				*Euthynnus lineatus*[b]		
									Auxis thazard[c]		
							B3K2	Sanger (-)*			
2021.03.08	21	C1A	愛情貴族	白身鮪魚&牛肉	白身鮪魚	Thailand	C1A1	Sanger (+)	*Katsuwonus pelamis*	No	Supplementary Information 2
									Thunnus albacares[a]		
					牛肉		C1A2	Sanger (-)*			
	22	C1B	愛情貴族	白身鮪魚&吻仔魚	白身鮪魚	Thailand	C1B1	Sanger (+)	*Katsuwonus pelamis*	No	Supplementary Information 2
					吻仔魚				*Thunnus albacares*[a]		
							C1B2	Sanger (-)*			
	23	C1C	愛情貴族	白身鮪魚&雞肉	白身鮪魚	Thailand	C1C1	Sanger (-)*		No	
					雞肉		C1C2	Sanger (+)	*Gallus gallus*		Supplementary Information 2

24	C1D	愛情貴族	白身鮪魚&鮭魚	白身鮪魚	Thailand	C1D1	Sanger (+)	*Katsuwonus pelamis*	No	Supplementary Information 2
				鮭魚				*Thunnus albacares*[a]		
						C1D2	Sanger (+)	*Oncorhynchus nerka*		Supplementary Information 2
25	C1E	愛情貴族	白身鮪魚&蟹肉	白身鮪魚	Thailand	C1E1	Sanger (+)	*Katsuwonus pelamis*	No	Supplementary Information 2
				蟹肉				*Thunnus albacares*[a]		
						C1E2	Sanger (+)	*Katsuwonus pelamis*		Supplementary Information 2
								Thunnus albacares[a]		
26	C1F	愛情貴族	白身鮪魚&柴魚	白身鮪魚	Thailand	C1F1	Sanger (+)	*Katsuwonus pelamis*	Yes	Supplementary Information 2
				柴魚				*Thunnus albacares*[a]		
						C1F2	Sanger (+)	*Auxis rochei*		Supplementary Information 2
								Auxis thazard		
								Euthynnus affinis[d]		

2021.03.11	27	C2A	每日貓罐	每日貓罐-雞肉+吻仔魚湯罐	雞肉	Taiwan	C2A1	Sanger (+)	*Gallus gallus*	Yes	Supplementary Information 2
					吻仔魚		C2A2	Sanger (+)	*Pholis fangi*		Supplementary Information 2
									Dictyosoma burgeri[e]		
	28	C2B	每日貓罐	每日貓罐-鮪魚+蟹味絲湯罐	鮪魚	Taiwan	C2B1	Sanger (+)	*Thunnus tonggol*	No	Supplementary Information 2
					蟹味絲				*Thunnus orientalis*		
									Thunnus obesus		
							C2B2	Sanger (-)*			
	29	C2C	每日貓罐	每日貓罐-鮪魚+巴沙魚湯罐	鮪魚	Taiwan	C2C1	Sanger (+)	*Thunnus tonggol*	No	Supplementary Information 2
					巴沙魚				*Thunnus orientalis*		
									Thunnus obesus		
							C2C2	Sanger (+)	*Pangasianodon hypophthalmus*		Supplementary Information 2
									Pangasius bocourti		
									Pangasius djambal		
									Pangasius sanitwongsei		

	30	C2D	每日貓罐	每日貓罐-雞肉+柴魚湯罐		雞肉	Taiwan	C2D1	Sanger (+)	*Gallus gallus*	No	Supplementary Information 2
						柴魚		C2D2	Sanger (-)*			
2021.03.23	31	C3A	CANIDAE	無穀主食罐(鮭魚、鮪魚、鯛魚湯罐)	Salmon & whitefish in gravy	鮭魚	Thailand	C3A1	Sanger (+)	*Oncorhynchus gorbuscha*	No	Supplementary Information 2
						鯛魚		C3A2	Sanger (+)	*Sparus aurata*		Supplementary Information 2
						鮪魚		C3A3	Sanger (+)	*Katsuwonus pelamis*		Supplementary Information 2
										Thunnus albacares[a]		
	32	C3B	CANIDAE	無穀主食罐(沙丁魚、鯖魚湯罐)	Chunky sardine & mackerel in broth	沙丁魚	Thailand	C3B1	Sanger (+)	*Sardinella fijiensis*	No	Supplementary Information 2
						魷魚				*Sardinella gibbosa*		
										Sardinella jussieu		
										Sardinella fimbriata		
								C3B2	Sanger (-)*			
	33	C3C	Applaws	愛普士全天然鮮食罐(鯖魚、雞胸肉)	Mackerel with chicken breast	鯖魚	Thailand	C3C1	Sanger (+)	*Scomber japonicus*	No	Supplementary Information 2
						雞胸肉				*Scomber scombrus*		
										Scomber australasicus		

										Scomber colias		
								C3C2	Sanger (+)	*Gallus gallus*		Supplementary Information 2
	34	C3D	Applaws	愛普士全天然鮮食罐(鮪魚)	Tuna fillet	鮪魚	Thailand	C3D	Sanger (+)	*Auxis rochei*	Yes	Supplementary Information 2
										Auxis thazard		
										Euthynnus affinis[d]		
	35	C3E	Applaws	愛普士全天然鮮食罐(鯖魚、沙丁魚)	Mackerel with sardine	鯖魚	Thailand	C3E1	Sanger (+)	*Scomber japonicus*	No	Supplementary Information 2
						沙丁魚				*Scomber scombrus*		
										Scomber australasicus		
										Scomber colias		
								C3E2	Sanger (+)	*Scomber japonicus*		Supplementary Information 2
										Scomber scombrus		
										Scomber australasicus		
										Scomber colias		
2021.03.26	36	C4A	鼎食貓罐	鼎食貓罐(新鮮鮪魚+丁香魚)		鮪魚	Taiwan	C4A1	Sanger (+)	*Thunnus tonggol*	Yes	Supplementary Information 2
						丁香魚				*Thunnus orientalis*		

										Thunnus atlanticus		
								C4A2	Sanger (+)	*Encrasicholina punctifer*		Supplementary Information 2
	37	C4B	鼎食貓罐	鼎食貓罐(旗魚)		旗魚	Taiwan	C4B	Sanger (-)*		Uncertain	
	38	C4C	鼎食貓罐	鼎食貓罐(新鮮鮪魚+櫻花蝦)		鮪魚	Taiwan	C4C1	Sanger (+)	*Thunnus tonggol*	No	Supplementary Information 2
						櫻花蝦				*Thunnus orientalis*		
										Thunnus atlanticus		
								C4C2	Sanger (+)	*Thunnus tonggol*		Supplementary Information 2
										Thunnus orientalis		
										Thunnus atlanticus		
	39	C4D	怪獸部落	無膠無穀鮮肉煲-鯖魚丁湯罐	Diced mackerel in broth	鯖魚	Thailand	C4D	Sanger (+)	*Scomber japonicus*	No	Supplementary Information 2
										Scomber scombrus		
										Scomber australasicus		
										Scomber colias		
2021.03.30	40	C5A	怪獸部落	無膠無穀鮮肉煲-鮪魚片湯罐	Flaked tuna in broth	鮪魚	Thailand	C5A	Sanger (+)	*Katsuwonus pelamis*	No	Supplementary Information 2

									Thunnus albacares[a]		
41	C5B	怪獸部落	無膠無穀鮮肉煲-鰹魚塊湯罐	Chopped skipjack in broth	鰹魚	Thailand	C5B	Sanger (+)	*Katsuwonus pelamis*	No	Supplementary Information 2
									Thunnus albacares[a]		
42	C5C	怪獸部落	無膠無穀先煲-嫩雞丁湯罐	Diced chicken in broth	雞肉	Thailand	C5C	Sanger (+)	*Gallus gallus*	No	Supplementary Information 2
43	C5G	Tiki Cat	夏日 5 號	Wild salmon recipe in salmon consommé	鮭魚	Thailand	C5G	Sanger (+)	*Oncorhynchus gorbuscha*	No	Supplementary Information 2
44	C5H	Tiki Cat	夏日 6 號	Ahi tuna & mackerel recipe in tuna consommé	鮪魚	Thailand	C5H1	Sanger (-)*		Uncertain	
					鯖魚		C5H2	Sanger (-)*			
45	C5I	Tiki Cat	夏日 4 號	Sardine cutlets recipe in lobster consommé	沙丁魚	Thailand	C5I	Sanger (+)	*Sardinella fijiensis*	No	Supplementary Information 2
									Sardinella gibbosa		
									Sardinella jussieu		
									Sardinella fimbriata		

2021.03.31	46	C6A	海之味	海之味貓咪主食罐 ES5 旗魚+雞肉	旗魚	Thailand	C6A1	Sanger (+)	*Istiophorus albicans*	No	Supplementary Information 2
					雞肉				*Istiophorus platypterus*		
					丁香魚				*Istiompax indica*		
					鮮蝦仁				*Makaira nigricans*		
					干貝				*Makaira mazara*		
					吻仔魚		C6A2	Sanger (+)	*Gallus gallus*		Supplementary Information 2
							C6A3	Sanger (+)	*Encrasicholina punctifer*		Supplementary Information 2
							C6A4	Sanger (-)*			
							C6A5	Sanger (-)*			
							C6A6	Sanger (+)	*Encrasicholina heteroloba*		Supplementary Information 2
	47	C6B	海之味	海之味貓咪主食罐 ES4 鮪魚+雞肉	鮪魚	Taiwan	C6B1	Sanger (-)*		Uncertain	
					雞肉		C6B2	Sanger (-)*			
					丁香魚		C6B3	Sanger (-)*			
					鮮蝦仁		C6B4	Sanger (-)*			
					干貝		C6B5	Sanger (-)*			
					吻仔魚		C6B6	Sanger (-)*			

	48	C6C	DELIZIOS	DELIZIOS 饕客貓湯罐 鰹魚佐頂級肉湯	鰹魚	Thailand	C6C	Sanger (-)*		Uncertain	
	49	C6D	DELIZIOS	DELIZIOS 饕客貓湯罐 鰹魚佐吻仔魚	鰹魚	Thailand	C6D1	Sanger (-)*		No	
					吻仔魚		C6D2	Sanger (+)	*Encrasicholina heteroloba*		Supplementary Information 2
	50	C6E	DELIZIOS	DELIZIOS 饕客貓湯罐 鰹魚塊+雕魚蝦肉佐肉湯	鰹魚	Thailand	C6E1	Sanger (-)*		Yes	
					鯛魚		C6E2	Sanger (+)	*Lates calcarifer*		Supplementary Information 2
					蝦				*Psammoperca waigiensis*[f]		
							C6E3	Sanger (-)*			
2021.04.14	51	D3A	好味小姐	1+1 貓鮮食主食罐(白醬嫩雞)	雞肉	Taiwan	D3A	Sanger (+)	*Gallus gallus*	No	Supplementary Information 2
	52	D3B	好味小姐	1+1 貓鮮食主食罐(白醬嫩雞)	牛肉	Taiwan	D3B1	Sanger (+)	*Bos taurus*	No	Supplementary Information 2
					雞肉		D3B2	Sanger (+)	*Gallus gallus*		Supplementary Information 2

	53	D3C	好味小姐	1+1 貓鮮食主食罐(白醬嫩雞)	鯛魚	Taiwan	D3C1	Sanger (+)	*Tilapia nilotica*	Yes	Supplementary Information 2
					雞肉		D3C2	Sanger (+)	*Gallus gallus*		Supplementary Information 2
					鮭魚		D3C3	Sanger (+)	*Gallus gallus*		Supplementary Information 2
2021.04.23	54	D1A	YAMI 亞米	YAMI 雞湯大餐 鮮雞.雞肝.青花魚	青花魚	Thailand	D1A1	Sanger (+)	*Gallus gallus*	No	Supplementary Information 2
					雞肉		D1A2	Sanger (-)*			
	55	D1B	YAMI 亞米	鮮鮪.雞肉白金大餐	鮪魚白肉	Thailand	D1B1	Sanger (+)	*Auxis thazard*	Yes	Supplementary Information 2
									Auxis rochei		
									Euthynnus affinis[d]		
					雞肉		D1B2	Sanger (+)	*Gallus gallus*		Supplementary Information 2
	56	D1C	YAMI 亞米	鮮鮪.青花魚.蟹柳白金大餐	鮪魚白肉	Thailand	D1C1	Sanger (+)	*Auxis thazard*	Yes	Supplementary Information 2
					青花魚				*Auxis rochei*		
					蟹柳				*Euthynnus affinis*[d]		

						D1C2	Sanger (+)	*Scomber japonicus*		Supplementary Information 2
								Scomber scombrus		
								Scomber australasicus		
								Scomber colias		
						D1C3	Sanger (-)*			
57	D1D	YAMI 亞米	YAMI 雞湯大餐 鮮雞.牛肉.鮭魚	雞肉	Thailand	D1D1	Sanger (+)	*Gallus gallus*	No	Supplementary Information 2
				鮭魚		D1D2	Sanger (+)	*Oncorhynchus gorbuscha*		Supplementary Information 2
				牛肉		D1D3	Sanger (+)	*Bos taurus*		Supplementary Information 2
58	D2A	TRIL GY	奇境 無穀貓罐 野生鯖魚燉雞湯	鯖魚	Thailand	D2A	Sanger (+)	*Scomber japonicus*	No	Supplementary Information 2
								Scomber scombrus		
								Scomber australasicus		
								Scomber colias		
59	D2B	TRIL GY	奇境 無穀貓罐 野生鮪魚燉雞湯	鮪魚	Thailand	D2B	Sanger (+)	*Katsuwonus pelamis*	No	Supplementary Information 2
								Thunnus albacares[a]		

2021.04.28	60	D2C	O'KAT	美喵人生 無穀化毛餐	鮪魚	Thailand	D2C1	Sanger (+)	*Katsuwonus pelamis*	No	Supplementary Information 2
									Thunnus albacares[a]		
					沙丁魚		D2C2	Sanger (+)	*Sardinella fijiensis*		Supplementary Information 2
									Sardinella gibbosa		
									Sardinella jussieu		
									Sardinella fimbriata		
	61	D2D	O'KAT	美喵人生 無穀超機能 好鮮肉罐	鯷魚	Thailand	D2D1	Sanger (-)*		No	
					鮪魚		D2D2	Sanger (+)	*Katsuwonus pelamis*		Supplementary Information 2
									Thunnus albacares[a]		
	62	D2E	O'KAT	美喵人生 無穀超機能 好濃肉罐	鯖魚	Thailand	D2E1	Sanger (-)*		No	
					蝦		D2E2	Sanger (+)	*Metapenaeopsis barbata*		Supplementary Information 2
									Metapenaeopsis palmensis		
									Metapenaeopsis stridulans		

	63	D2F	Rico	芮可-貓用副食鮮湯罐 4 號(鮪雞+吻仔魚)	鮪魚	Taiwan	D2F1	Sanger (-)*		No	
					雞肉		D2F2	Sanger (+)	*Gallus gallus*		Supplementary Information 2
					吻仔魚		D2F3	Sanger (+)	*Encrasicholina heteroloba*		Supplementary Information 2
	64	D2G	Rico	芮可-貓用副食鮮湯罐 2 號(鮪雞+鰹魚)	鰹魚	Taiwan	D2G1	Sanger (-)*		No	
					雞肉		D2G2	Sanger (+)	*Gallus gallus*		Supplementary Information 2
					鮪魚		D2G3	Sanger (+)	*Thunnus tonggol*		Supplementary Information 2
									Thunnus orientalis		
2021.05.05	65	E1A	Signature7	新寵七味賞 星期四 白身鮪魚+南瓜	白身鮪魚	Thailand	E1A	Sanger (-)*		Uncertain	
	66	E1B	Signature7	新寵七味賞 星期四 鯖魚+胡蘿蔔	鯖魚	Thailand	E1B	Sanger (-)*		Uncertain	
	67	E1C	Lungevity	永恆無穀主食罐	雞肉		E1C1	Sanger (+)	*Gallus gallus*	No	Supplementary Information 2

					白鰹魚	Thailand	E1C2	Sanger (-)*			
	68	E1D	湯貓道	湯貓道之無膠營養上湯罐	白身鮪魚	Taiwan	E1D1	Sanger (-)*		No	
					雞肉		E1D2	Sanger (+)	*Gallus gallus*		Supplementary Information 2
					吻仔魚		E1D3	Sanger (-)*			
	69	E1E	AIXIA	樂妙喵 3 號-鮪.吻仔	鮪魚	Japan	E1E1	Sanger (+)	*Thunnus tonggol*	No	Supplementary Information 2
									Thunnus orientalis		
					吻仔魚		E1E2	Sanger (+)	*Encrasicholina punctifer*		Supplementary Information 2
	70	E1F	AIXIA	樂妙喵 4 號-鮪.柴魚	鮪魚	Japan	E1F1	Sanger (+)	*Thunnus tonggol*	No	Supplementary Information 2
									Thunnus orientalis		
					柴魚		E1F2	Sanger (-)*			
2021.05.10	71	E2A	厚肉肉	T.N.A 悠遊厚肉肉主食罐.海味旗魚拚海瓜子	旗魚	Taiwan	E2A	Sanger (+)	*Istiophorus albicans*	No	Supplementary Information 2
									Istiophorus platypterus		
									Istiompax indica		

								Makaira nigricans		
								Makaira mazara		
72	E2B	厚肉肉	T.N.A 悠遊厚肉肉主食罐-至尊鰹魚拚吻仔魚	鰹魚	Taiwan	E2B1	Sanger (-)*		No	
				吻仔魚		E2B2	Sanger (+)	*Encrasicholina punctifer*		Supplementary Information 2
73	E2C	厚肉肉	T.N.A 悠遊厚肉肉主食罐-一品鮪魚拚鮭魚	鮪魚	Taiwan	E2C	Sanger (+)	*Thunnus tonggol*	No	Supplementary Information 2
								Thunnus orientalis		
74	E2D	AIXIA	燒津 43 號-鮪.雞.鮭	鮪魚	Japan	E2D1	Sanger (+)	*Thunnus tonggol*	No	Supplementary Information 2
								Thunnus orientalis		
				雞肉		E2D2	Sanger (+)	*Gallus gallus*		Supplementary Information 2
				鮭魚		E2D3	Sanger (+)	*Oncorhynchus nerka*		Supplementary Information 2
75	E2E	愛喜雅	燒津 42 號-鮪.雞.吻	鮪魚	Japan	E2E1	Sanger (+)	*Thunnus tonggol*	No	Supplementary Information 2
								Thunnus orientalis		
				雞肉		E2E2	Sanger (-)*			

					吻仔魚		E2E3	Sanger (+)	*Encrasicholina heteroloba*		Supplementary Information 2
	76	C5F	小怪獸	怪獸部落 2 種肉無膠主食罐-貓用鱉肉雞肉餐	雞肉	Taiwan	C5F1	Sanger (-)*		No	
					鱉肉		C5F2	Sanger (+)	*Gallus gallus*		Supplementary Information 2
					鰹魚						
2021.05.14	77	E3A	CIAO	多樂米濃湯罐(雞肉+鰹魚+吻仔魚)	雞肉	Japan	E3A1	Sanger (+)	*Gallus gallus*	No	Supplementary Information 2
					鰹魚		E3A2	Sanger (+)	*Gallus gallus*		Supplementary Information 2
					吻仔魚		E3A3	Sanger (-)*			
	78	E3B	CIAO	多樂米濃湯罐(鮪魚+雞肉+柴魚片)	鮪魚(鰹魚)	Japan	E3B1	Sanger (+)	*Gallus gallus*	No	Supplementary Information 2
					雞肉		E3B2	Sanger (+)	*Gallus gallus*		Supplementary Information 2
					柴魚片		E3B3	Sanger (+)	*Thunnus obesus*		Supplementary Information 2
									Thunnus tonggol		

79	E3C	N&D	N&D 挑嘴成貓天然海洋主食罐-鮪魚蝦肉	Tuna & shrimp	鮪魚	Serbia	E3C1	Sanger (+)	*Thunnus tonggol*	No	Supplementary Information 2
									Thunnus orientalis		
					蝦肉		E3C2	Sanger (-)*			
80	E3D	Hagoromo	有魚魚真幸福貓罐頭-吻仔魚添加		鮪魚	Japan	E3D1	Sanger (+)	*Thunnus tonggol*	No	Supplementary Information 2
									Thunnus orientalis		
					吻仔魚		E3D2	Sanger (+)	*Encrasicholina heteroloba*		Supplementary Information 2
81	E3E	Cat's Voice	日本 VOICE 五星頂級貓罐 鮪魚+魚子醬		鮪魚	Japan	E3E1	Sanger (+)	*Thunnus tonggol*	No	Supplementary Information 2
									Thunnus orientalis		
					魚子醬		E3E2	Sanger (+)	*Acipenser fulvescens*		Supplementary Information 2
									Acipenser gueldenstaedtii		
									Huso dauricus		
									Acipenser brevirostrum		
									Acipenser mikadoi		
									Acipenser medirostris		
									Acipenser ruthenus		
									Acipenser baerii		

										Acipenser naccarii		
										Acipenser baerii baicalensis		
2021.05.31	82	E4A	Sheba	SHEBA 金罐 白身鮪魚及鯛魚(湯汁)	Tuna with bream in jelly	鮪魚	Thailand	E4A1	Sanger (+)	*Thunnus obesus*	No	Supplementary Information 2
										Thunnus tonggol		
						鯛魚		E4A2	Sanger (+)	*Priacanthus macracanthus*		Supplementary Information 2
	83	E4B	Sheba	SHEBA 金罐 白身鮪魚及魴魚(魚凍)	Tuna with bream in jelly	鮪魚	Thailand	E4B1	Sanger (+)	*Thunnus tonggol*	No	Supplementary Information 2
										Thunnus orientalis		
						白身魚		E4B2	Sanger (+)	*Nemipterus zysron*		Supplementary Information 2
						魴魚		E4B3	Sanger (-)*			
	84	E4C	17MEOW	17 喵貓罐-鮪魚+鯛魚	Tuna + Seabream	鮪魚	Vietnam	E4C1	Sanger (+)	*Katsuwonus pelamis*	No	Supplementary Information 2
										Thunnus albacares[a]		
						鯛魚		E4C2	Sanger (-)*			
	85	E4D	汪喵星球	貓咪點心罐 白身鮪魚綠貽貝		鮪魚	Thailand	E4D1	Sanger (+)	*Katsuwonus pelamis*	No	Supplementary Information 2

						綠貽貝		E4D2	Sanger (+)	*Thunnus albacares*[a]<br>*Thunnus obesus*<br>*Thunnus tonggol*		Supplementary Information 2
	86	E4E	汪喵星球	貓咪點心罐 鰹魚蛤蠣小雙拼		鰹魚	Thailand	E4E1	Sanger (+)	*Katsuwonus pelamis*	Yes	Supplementary Information 2
						蛤蠣		E4E2	Sanger (+)	*Thunnus albacares*[a]<br>*Thunnus obesus*<br>*Thunnus tonggol*		Supplementary Information 2
	87	F1E	靖禾風貓食	靖禾風貓食特級米罐(鮪魚+米+鮭魚)		鮪魚	Thailand	F1E1	Sanger (+)	*Katsuwonus pelamis*<br>*Thunnus albacares*[a]	No	Supplementary Information 2
						鮭魚		F1E2	Sanger (-)*			
2021.06.25	88	F4A	邦比-貓餐罐	邦比-貓餐罐 白身鮪魚+吻仔魚	Tuna & shirasu	白身鮪魚	Thailand	F4A1	Sanger (+)	*Katsuwonus pelamis*<br>*Thunnus albacares*[a]	No	Supplementary Information 2
						吻仔魚		F4A2	Sanger (+)	*Encrasicholina heteroloba*		Supplementary Information 2

89	F4B	藏鮮	藏鮮營養貓餐罐-鮪魚+鮭魚	Tuna & salmon	鮪魚	Taiwan	F4B1	Sanger (-)*		Uncertain	
					鮭魚		F4B2	Sanger (-)*			
90	F4C	NatureKE	紐崔克/貓罐 鮪魚+吻仔魚(凍罐)	Tuna & shirasu	鮪魚	Taiwan	F4C1	Sanger (+)	*Thunnus tonggol*	No	Supplementary Information 2
									Thunnus atlanticus		
									Thunnus alalunga		
									Thunnus albacares		
									Thunnus maccoyii		
					吻仔魚		F4C2	Sanger (+)	*Encrasicholina punctifer*		Supplementary Information 2
91	F4D	CATs happy day	幸福時光-貓營養主食 2 號罐(雞肉+鮪魚+巴沙魚)	Chicken & tuna & bassafish	雞肉	Vietnam	F4D1	Sanger (+)	*Gallus gallus*	No	Supplementary Information 2
					鮪魚		F4D2	Sanger (+)	*Thunnus tonggol*		Supplementary Information 2
									Thunnus atlanticus		
									Thunnus alalunga		
									Thunnus albacares		
									Thunnus maccoyii		

						巴沙魚		F4D3	Sanger (+)	*Pangasianodon hypophthalmus*		Supplementary Information 2
2021.07.05	92	F5A	義士大廚	鮪魚鮮嫩罐-鮪魚吻仔魚		鮪魚	Thailand	F5A1	Sanger (+)	*Auxis thazard*	Yes	Supplementary Information 2
										Auxis rochei		
										Euthynnus affinis[d]		
						吻仔魚		F5A2	Sanger (+)	*Sardinella fijiensis*		Supplementary Information 2
										Sardinella jussieu		
										Sardinella fimbriata		
	93	F5B	日清	日清極品貓罐	Skipjack & salmon	鰹魚	Thailand	F5B1	Sanger (+)	*Katsuwonus pelamis*	No	Supplementary Information 2
										Thunnus albacares[a]		
						鮭魚		F5B2	Sanger (+)	*Oncorhynchus nerka(23)*		Supplementary Information 2
	94	F5C	日清	日清極品貓罐	Yellowfin & shirasu	鮪魚	Thailand	F5C1	Sanger (+)	*Thunnus tonggol*	No	Supplementary Information 2
										Thunnus atlanticus		
										Thunnus alalunga		
										Thunnus albacares		

									Thunnus maccoyii		
					吻仔魚		F5C2	Sanger (+)	*Engraulis japonicus*		Supplementary Information 2
	95	F5D	CARNY	德國阿曼達 ANIMONDA 卡恩海洋貓罐	鮭魚	Thailand	F5D1	Sanger (+)	*Oncorhynchus gorbuscha*	No	Supplementary Information 2
					吻仔魚		F5D2	Sanger (+)	*Encrasicholina heteroloba*		Supplementary Information 2
2021.07.14	96	F5E	CARNY	德國阿曼達 ANIMONDA 卡恩海洋貓罐	鮪魚	Thailand	F5E1	Sanger (+)	*Katsuwonus pelamis*<br>*Thunnus albacares*[a]	No	Supplementary Information 2
					鯛魚		F5E2	Sanger (+)	*Lutjanus malabaricus*<br>*Lutjanus erythropterus*		Supplementary Information 2
	97	F6A	me-o	咪歐小確幸-蔬菜鮪魚	鮪魚	Thailand	F6A	Sanger (+)	*Katsuwonus pelamis*<br>*Thunnus albacares*[a]	No	Supplementary Information 2

	98	F6B	AIXIA	黑金罐主食 2 號鰹.鮪.魩	鰹魚	Korea	F6B1	Sanger (+)	*Katsuwonus pelamis*<br>*Thunnus albacares*[a]	No	Supplementary Information 2
					鮪魚		F6B2	Sanger (+)	*Katsuwonus pelamis*<br>*Thunnus albacares*[a]		Supplementary Information 2
					吻仔魚		F6B3	Sanger (+)	*Katsuwonus pelamis*<br>*Thunnus albacares*[a]		Supplementary Information 2
2021.07.15	99	G1A	偉嘉	偉嘉貓食溼糧 海洋大餐	鮪魚	Thailand	G1A1	Sanger (+)	*Katsuwonus pelamis*<br>*Thunnus albacares*[a]	No	Supplementary Information 2
					沙丁魚		G1A2	Sanger (+)	*Katsuwonus pelamis*<br>*Thunnus albacares*[a]		Supplementary Information 2
					鯖魚		G1A3	Sanger (-)*			
2021.09.13	100	I1A	CIAO	CIAO 旨定罐 雞肉+烤鰹魚 貓用	雞肉	Japan	I1A1	Sanger (+)	*Gallus gallus*	No	Supplementary Information 2
					鰹魚		I1A2	Sanger (+)	*Katsuwonus pelamis*<br>*Thunnus albacares*[a]		Supplementary Information 2

	101	I1B	OASY	鮪魚佐魷魚鮮食	Tuna with squid	鮪魚	Thailand	I1B1	Sanger (+)	*Katsuwonus pelamis*	No	Supplementary Information 2
										Thunnus albacares[a]		
						魷魚		I1B2	Sanger (-)*			
	102	I1C	快樂尾	貓肯罐 護心鮭魚雞泥		雞肉	Taiwan	I1C1	Sanger (-)*		Uncertain	
						鮭魚		I1C2	Sanger (-)*			
2021.09.17	103	I2A	Gim Cat	化毛貓罐-鮪魚	Tonno Tuna	鮪魚	Thailand	I2A	Sanger (-)*		Uncertain	
	104	I2B	Eats	易特廚 吻仔魚精緻大餐		鮪魚	Thailand	I2B1	Sanger (+)	*Katsuwonus pelamis*	No	Supplementary Information 2
										Thunnus albacares[a]		
						雞肉		I2B2	Sanger (+)	*Gallus gallus*		Supplementary Information 2
						吻仔魚		I2B3	Sanger (+)	*Encrasicholina punctifer*		Supplementary Information 2
	105	I2C	Dr. Clauder's	3 號鮪魚雞肉	Tuna & chicken breast	鮪魚	Thailand	I2C1	Sanger (-)*		No	
						雞肉		I2C2	Sanger (+)	*Gallus gallus*		Supplementary Information 2

	106	I2D	Dr. Clauder's	4 號鮪魚鮭魚	Tuna & salmon	鮪魚	Thailand	I2D1	Sanger (+)	*Katsuwonus pelamis*	No	Supplementary Information 2
										Thunnus albacares[a]		
						鮭魚		I2D2	Sanger (+)	*Oncorhynchus gorbuscha*		Supplementary Information 2
2021.11.12	107	J1A	Cherie 法麗	室內貓晝毛配方 微湯汁鮪魚佐鮭魚	Tuna topping salmon in gravy	鮪魚	Thailand	J1A1	Sanger (+)	*Katsuwonus pelamis*	No	Supplementary Information 2
										Thunnus albacares[a]		
						鮭魚	Thailand	J1A2	Sanger (+)	*Oncorhynchus gorbuscha*		Supplementary Information 2
	108	J1B	Cherie 法麗	微湯汁系列 天然黃鰭鮪佐正鰹、鮭魚	Flaked yellowfin mix, skipjack tuna with wild salmon entrée in Gravy	黃鰭鮪魚	Thailand	J1B1	Sanger (+)	*Katsuwonus pelamis*	No	Supplementary Information 2
										Thunnus albacares[a]		
						鰹魚	Thailand	J1B2	Sanger (+)	*Oncorhynchus gorbuscha*		Supplementary Information 2
						鮭魚	Thailand	J1B3	Sanger (+)	*Oncorhynchus gorbuscha*		Supplementary Information 2
	109	J1C	Cherie 法麗	微湯汁系列 天然黃鰭鮪佐正鰹	Flaked yellowfin mix skipjack tuna entrée in gravy	黃鰭鮪魚	Thailand	J1C1	Sanger (+)	*Katsuwonus pelamis*	No	Supplementary Information 2

									Thunnus albacares[a]		
					鰹魚	Thailand	J1C2	Sanger (+)	*Thunnus tonggol*		Supplementary Information 2
									Thunnus atlanticus		
									Thunnus alalunga		
									Thunnus albacares		
									Thunnus maccoyii		
	110	J1D	Natural kitty	無膠肉湯罐-鮪魚佐鯷魚魚湯	鮪魚	Thailand	J1D1	Sanger (+)	*Katsuwonus pelamis*	No	Supplementary Information 2
									Thunnus albacares[a]		
					鯷魚	Thailand	J1D2	Sanger (+)	*Encrasicholina punctifer*		Supplementary Information 2
2021.11.17	111	J2A	CIAO	幼貓罐鮪魚+鰹魚 1 歲以下	鮪魚	Japan	J2A1	Sanger (+)	*Thunnus tonggol*	No	Supplementary Information 2
									Thunnus atlanticus		
									Thunnus alalunga		
									Thunnus albacares		
									Thunnus maccoyii		
					吻仔魚		J2A2	Sanger (+)	*Engraulis japonicus*		Supplementary Information 2

112	J2B	PLAN CAT	喵洽普貓罐-鰆魚&吻仔魚	Tuna & whitebait	鰆魚	Thailand	J2B1	Sanger (+)	*Katsuwonus pelamis*	No	Supplementary Information 2
									Thunnus albacares[a]		
					吻仔魚		J2B2	Sanger (+)	*Encrasicholina punctifer*		Supplementary Information 2
113	J2C	Eats 吃	易特廚 奢華嫩鮭魚大餐(鮪魚+雞肉+鮭魚)		鮪魚	Thailand	J2C1	Sanger (-)*		No	
					雞肉		J2C2	Sanger (+)	*Gallus gallus*		Supplementary Information 2
					鮭魚						
114	I3B	快樂尾	貓肯罐 雙魚複合保養		鮪魚	Taiwan	I3B1	Sanger (-)*		No	
					旗魚		I3B2	Sanger (+)	*Istiophorus albicans*		Supplementary Information 2
									Istiophorus platypterus		
									Istiompax indica		
									Makaira nigricans		
									Makaira mazara		
115	I3C	銀湯匙	貓罐頭雞肉+吻仔魚		鰹魚	Thailand	I3C1	Sanger (+)	*Gallus gallus*	No	Supplementary Information 2
					鮪魚		I3C2	Sanger (-)*			

						吻仔魚		I3C3	Sanger (-)*			
						雞肉		I3C4	Sanger (+)	*Gallus gallus*		Supplementary Information 2
	116	I3D	銀湯匙	貓罐頭鮪魚+鰹魚+吻仔魚		鮪魚	Thailand	I3D1	Sanger (+)	*Katsuwonus pelamis*	No	Supplementary Information 2
										*Thunnus albacares**		
						鰹魚		I3D2	Sanger (+)	*Katsuwonus pelamis*		Supplementary Information 2
										*Thunnus albacares**		
2021.12.14	117	L1A	Applaws	愛普士全天然主食罐(鮪魚-幼貓配方)	Kitten-tuna in jelly	鮪魚	Thailand	L1A	Sanger (+)	*Auxis thazard*	Yes	Supplementary Information 2
										Auxis rochei		
										Euthynnus affinis[d]		
	118	B2C	SEEDS	Dr. Wish 愛貓調整配方營養食(泥狀)	Tuna + chicken + vitamin B complex	鮪魚	Thailand	B2C	NGS (+)	*Thunnus alalunga*	Yes	GenBank: PRJNA1036020
						雞肉				*Thunnus atlanticus*		
										Thunnus obesus		
										Katsuwonus pelamis		
										Auxis rochei		
										Auxis thazard		

								Euthynnus affinis		
								Sarda orientalis		
								Micromesistius sp.		
								Gallus gallus		
								Bos taurus		
								Cervus elaphus		
								Ovis aries		
								Felis catus		
								Homo sapiens		
								Trachypenaeus curvirostris		
								Plicofollis nella		
								Acinetobacter junii		
119	I3A	快樂尾	貓肯罐 戶心雞鮪鮮泥	鮪魚	Taiwan	I3A	NGS (+)	*Thunnus alalunga*	Yes	GenBank: PRJNA1036020
				雞肉				*Thunnus atlanticus*		
								Thunnus obesus		
								Katsuwonus pelamis		
								Auxis thazard		
								Scomber colias		
								Cololabis saira		
								Oncorhynchus gorbuscha		
								Salmo environmental sample		

										Sardinella fijiensis		
										Chanos chanos		
										Gallus gallus		
										Meleagris gallopavo		
										Homo sapiens		
2021.06.07	120	F2A	GAT-POOL	雞肉鮪魚干貝主食罐	Chicken, tuna & scallop	雞肉	Taiwan	F2A	NGS (+)	*Gallus gallus*	Yes	GenBank: PRJNA1036020
						鮪魚				*Thunnus alalunga*		
						干貝				*Thunnus atlanticus*		
						鱉蛋粉				*Thunnus obesus*		
										Katsuwonus pelamis		
										Allothunnus fallai		
										Auxis rochei		
										Auxis thazard		
										Euthynnus affinis		
										Sarda orientalis		
										Pelodiscus sinensis		
										Isurus oxyrinchus		
										Homo sapiens		
										Bos taurus		
										Trachypenaeus curvirostris		

	121	F2B	PURRFACT	波菲特貓用主食罐(無加膠)	Chicken, fish & chicken broth	雞肉	Taiwan	F2B	NGS (+)	*Gallus gallus*	Yes	GenBank: PRJNA1036020
						鰹魚				*Coturnix japonica*		
						魚油				*Katsuwonus pelamis*		
										Auxis thazard		
										Auxis rochei		
										Euthynnus affinis		
										Thunnus obesus		
										Thunnus atlanticus		
										Thunnus alalunga		
										Istiophorus platypterus		
										Homo sapiens		
										Trachypenaeus curvirostris		
2021.06.10	122	F2C	ZIWI	鯖魚羊肉主食罐	NZ Mackerel & lamb recipe	鯖魚	New Zealand	F2C	NGS (+)	*Thunnus obesus*	Yes	GenBank: PRJNA1036020
						羊肉				*Thunnus atlanticus*		
						綠唇貝				*Thunnus alalunga*		
										Katsuwonus pelamis		
										Sarda orientalis		
										Auxis thazard		
										Auxis rochei		
										Euthynnus affinis		

								Scomber colias		
								Scomber australasicus		
								Capra hircus		
								Ovis canadensis		
								Ovis aries		
								Ovis orientalis		
								Ovis ammon		
								Cervus elaphus		
								Cervus hanglu		
								Bos taurus		
								Homo sapiens		
								Trachypenaeus curvirostris		
								Cutibacterium acnes		
123	F2D	超越汪喵	雞肉+鰹魚+蝦仁大餐 (貓用)	雞肉	Taiwan	F2D	NGS (+)	*Gallus gallus*	Yes	GenBank: PRJNA1036020
				鰹魚				*Anas platyrhynchos*		
				蝦仁				*Katsuwonus pelamis*		
				魚油				*Auxis thazard*		
								Auxis rochei		
								Euthynnus affinis		
								Sarda orientalis		
								Allothunnus fallai		

									Thunnus obesus		
									Thunnus alalunga		
									Thunnus atlanticus		
									Cervus elaphus		
									Homo sapiens		
									Trachypenaeus curvirostris		
124	F2E	汪喵星球	貓咪雞肉鰹魚蝦仁餐	Chicken, bonito & shrimp	雞肉	Taiwan	F2E	NGS (+)	*Gallus gallus*	Yes	GenBank: PRJNA1036020
					鰹魚				*Katsuwonus pelamis*		
					黃金蜆				*Auxis thazard*		
					蝦仁				*Auxis rochei*		
					魚油				*Euthynnus affinis*		
									Thunnus atlanticus		
									Thunnus obesus		
									Thunnus alalunga		
									Sarda orientalis		
									Sus scrofa		
									Homo sapiens		
									Acinetobacter junii		
125	F2F	M'DARYN 肉食控	喵樂肉食控主食罐(嫩雞鮪魚)		雞肉	Taiwan	F2F	NGS (+)	*Gallus gallus*	Yes	GenBank: PRJNA1036020

					鮪魚				*Thunnus obesus*		
									Thunnus alalunga		
									Thunnus atlanticus		
									Katsuwonus pelamis		
									Auxis thazard		
									Euthynnus affinis		
									Sarda orientalis		
									Salmo environmental sample		
									Felis catus		
									Homo sapiens		
2021.06.15	126	F3A	M'DARYN 肉食控	喵樂肉食控主食罐(嫩雞旗魚)	雞肉	Taiwan	F3A	NGS (+)	*Gallus gallus*	Yes	GenBank: PRJNA1036020
					旗魚				*Makaira indica*		
									Thunnus alalunga		
									Thunnus obesus		
									Thunnus atlanticus		
									Katsuwonus pelamis		
									Auxis thazard		
									Auxis rochei		
									Euthynnus affinis		
									Sarda orientalis		
									Istiophorus platypterus		

								Sus scrofa		
								Ovis aries		
								Homo sapiens		
								Trachypenaeus curvirostris		
127	F3C	ZIWI Peak	ziwi peak 巔峰鮮肉貓罐頭-奧塔哥山谷牛鹿	牛肉	New Zealand	F3C	NGS (+)	*Bos gaurus*	Yes	GenBank: PRJNA1036020
				鹿肉				*Bos taurus*		
				蘭鱈魚				*Cervus elaphus*		
				鱈魚				*Capra hircus*		
				羊肚				*Cervus canadensis*		
				綠唇貝				*Cervus nippon*		
								Ovis canadensis		
								Ovis orientalis		
								Ovis ammon		
								Ovis aries		
								Micromesistius poutassou		
								Micromesistius sp.		
								Macruronus magellanicus		
								Thunnus atlanticus		
								Thunnus obesus		
								Thunnus alalunga		
								Katsuwonus pelamis		

								Auxis thazard		
								Auxis rochei		
								Euthynnus affinis		
								Sarda orientalis		
								Arripis truttaceus		
								Gallus gallus		
								Trachypenaeus curvirostris		
								Phascolosoma esculenta		
128	F3D	超越汪喵	深海鱈魚餐(貓用)	鱈魚	Taiwan	F3D	NGS (+)	*Atheresthes stomias*	Yes	GenBank: PRJNA1036020
				雞肉				*Thunnus alalunga*		
				魚油				*Thunnus obesus*		
								Thunnus atlanticus		
								Katsuwonus pelamis		
								Auxis thazard		
								Auxis rochei		
								Euthynnus affinis		
								Sarda orientalis		
								Gallus gallus		
								Coturnix japonica		
								Bos taurus		
								Ovis aries		

								Cervus elaphus		
								Homo sapiens		
								Propionibacterium acnes		
129	L1B	漁極	漁極 AM32 慕絲貓罐 (鮪+鮭)	鮪魚	Thailand	L1B	NGS (+)	*Thunnus alalunga*	Yes	GenBank: PRJNA1036020
				鮭魚				*Thunnus atlanticus*		
								Thunnus obesus		
								Katsuwonus pelamis		
								Allothunnus fallai		
								Auxis rochei		
								Auxis thazard		
								Euthynnus affinis		
								Oncorhynchus gorbuscha		
								Salmo environmental sample		
								Chanos chanos		
								Cololabis saira		
								Reinhardtius hippoglossoides		
								Scomber colias		
								Gallus gallus		
								Meleagris gallopavo		
								Meleagris ocellata		
								Bos taurus		

130	L1C	AIXIA	健康罐幼貓 6 號-雞、鮪	雞肉<br>鮪魚	Thailand	L1C	NGS (+)	*Thunnus alalunga*<br>*Thunnus albacares*<br>*Thunnus atlanticus*<br>*Thunnus obesus*<br>*Katsuwonus pelamis*<br>*Allothunnus fallai*<br>*Auxis rochei*<br>*Auxis thazard*<br>*Euthynnus affinis*<br>*Scomber colias*<br>*Salmo* environmental sample<br>*Oncorhynchus gorbuscha*<br>*Chanos chanos*<br>*Cololabis saira*<br>*Reinhardtius hippoglossoides*<br>*Gallus gallus*<br>*Meleagris gallopavo*<br>*Meleagris ocellata*<br>*Homo sapiens*	Yes	GenBank: PRJNA1036020

	131	L1D	WELLNESS	SS 名廚特選主食罐(貓用)鮮鮪魚泥+鮮鮭魚泥	Skipjack tuna & wild salmon Entrée	鮪魚	Thailand	L1D	NGS (+)	*Thunnus alalunga*	Yes	GenBank: PRJNA1036020
						鮭魚				*Thunnus atlanticus*		
										Thunnus obesus		
										Katsuwonus pelamis		
										Auxis thazard		
										Euthynnus affinis		
										Scomber colias		
										Oncorhynchus gorbuscha		
										Salmo environmental sample		
										Chanos chanos		
										Cololabis saira		
										Encrasicholina punctifer		
										Gallus gallus		
										Meleagris gallopavo		
										Homo sapiens		
										Acinetobacter junii		
2021.12.21	132	L2A	Hero MAMA	HeroMama 貓咪朔源鮮肉主食罐(虱目魚)		虱目魚	Taiwan	L2A	NGS (+)	*Chanos chanos*	Yes	GenBank: PRJNA1036020
										Thunnus alalunga		
										Thunnus atlanticus		
										Thunnus obesus		

									Katsuwonus pelamis		
									Auxis thazard		
									Scomber colias		
									Cololabis saira		
									Salmo environmental sample		
									Reinhardtius hippoglossoides		
									Oncorhynchus gorbuscha		
									Gallus gallus		
									Meleagris gallopavo		
									Meleagris ocellata		
133	L2B	小怪獸	Litomon 怪獸部落 2 種肉無膠主食罐-貓用鯖魚雞肉餐	Mackerel & chicken recipe for cats	鯖魚	Taiwan	L2B	NGS (+)	*Thunnus alalunga*	Yes	GenBank: PRJNA1036020
					雞肉				*Thunnus atlanticus*		
									Thunnus obesus		
									Katsuwonus pelamis		
									Auxis thazard		
									Scomber colias		
									Chanos chanos		
									Cololabis saira		
									Lates calcarifer		
									Oncorhynchus gorbuscha		

									Salmo environmental sample		
									Gallus gallus		
									Meleagris gallopavo		
									Cervus elaphus		
134	L2C	怪獸部落	怪獸部落寶寶無膠主食罐-幼貓比目魚		雞肉	Taiwan	L2C	NGS (+)	*Gallus gallus*	Yes	GenBank: PRJNA1036020
					鰹魚				*Meleagris gallopavo*		
					比目魚				*Katsuwonus pelamis*		
									Auxis thazard		
									Thunnus alalunga		
									Thunnus atlanticus		
									Thunnus obesus		
									Reinhardtius hippoglossoides		
									Scomber colias		
									Oncorhynchus gorbuscha		
									Salmo environmental sample		
									Chanos chanos		
									Cololabis saira		
135	L2D	挑嘴罐	汪喵星球挑嘴貓主食罐	Sauryfish & chicken	鰹魚	Taiwan	L2D	NGS (+)	*Katsuwonus pelamis*	Yes	GenBank: PRJNA1036020
					秋刀魚				*Thunnus alalunga*		

						雞肉				*Thunnus atlanticus*		
										Thunnus obesus		
										Allothunnus fallai		
										Auxis thazard		
										Euthynnus affinis		
										Scomber colias		
										Cololabis saira		
										Oncorhynchus gorbuscha		
										Reinhardtius hippoglossoides		
										Salmo environmental sample		
										Chanos chanos		
										Engraulis japonicus		
										Encrasicholina punctifer		
										Gallus gallus		
										Meleagris gallopavo		
										Meleagris ocellata		
										Sus scrofa		
										Bos taurus		
										Cervus elaphus		
										Homo sapiens		
2021.12.23	136	L3A	德國竣寶	營養幼貓罐-鮪魚	Tuna	鮪魚	Thailand	L3A	NGS (+)	*Thunnus alalunga*	Yes	GenBank: PRJNA1036020

								Thunnus atlanticus		
								Thunnus obesus		
								Katsuwonus pelamis		
								Auxis rochei		
								Auxis thazard		
								Scomber colias		
								Euthynnus affinis		
								Chanos chanos		
								Reinhardtius hippoglossoides		
								Cololabis saira		
								Oncorhynchus gorbuscha		
								Salmo environmental sample		
								Engraulis japonicus		
								Encrasicholina punctifer		
								Gallus gallus		
								Meleagris gallopavo		
								Meleagris ocellata		
								Felis catus		
								Homo sapiens		
137	L3B	林克博士	低敏主食貓罐	火雞肉	German	L3B	NGS (+)	*Meleagris gallopavo*	Yes	GenBank: PRJNA1036020
				鮭魚				*Meleagris ocellata*		

								Gallus gallus		
								Salmo environmental sample		
								Oncorhynchus gorbuscha		
								Coregonus hoyi		
								Coregonus sardinella		
								Thunnus alalunga		
								Thunnus atlanticus		
								Thunnus obesus		
								Katsuwonus pelamis		
								Auxis thazard		
								Scomber colias		
								Chanos chanos		
								Cololabis saira		
								Bos taurus		
138	L3C	羅西納	肉凍主食貓罐 鮪魚+鮮蝦	鮪魚	German	L3C	NGS (+)	*Thunnus alalunga*	Yes	GenBank: PRJNA1036020
				鮮蝦				*Thunnus atlanticus*		
								Thunnus obesus		
								Katsuwonus pelamis		
								Auxis thazard		
								Scomber colias		
								Chanos chanos		

Cololabis saira
Engraulis japonicus
Salmo environmental sample
Coregonus sardinella
Oncorhynchus gorbuscha
Coregonus hoyi
Gallus gallus
Meleagris gallopavo
Meleagris ocellata
Anas platyrhynchos
Canis lupus
Sus scrofa
Bos taurus
Homo sapiens

†In brackets, “+” indicates successful Sanger sequencing, whereas “–“ indicates failure.

*Sanger sequencing could not be conducted due to unsuccessful PCR.

[a]KM055376 is very likely to be *Katsuwonus pelamis* rather than *Thunnus albacares* (Chang, Kao, et al., 2021)

[b]LN558771 is very likely to be *Euthynnus affinis* rather than *E. lineatus*.

[c]MW595783 is very likely to be *E. affinis* rather than *Auxis thazard*.

[d]KM198909 is very likely to be *A. thazard* or *A. rochei* rather than *E. affinis*.

[e]NC_053709 is very likely to be *Pholis fangi* rather than *Dictyosoma burgeri*.

[f]KM198901 is very likely to be *Lates calcarifer* rather than *Psammoperca waigiensis*.
