# Supplementary material for: DNA mini-barcoding reveals the mislabeling rate of canned cat food in Taiwan
Source: PeerJ. 2024 Feb 21;12:e16833. doi: 10.7717/peerj.16833 (PMC10893872; doi:10.7717/peerj.16833)
Supplement: Supplemental Information 2 [file peerj-12-16833-s002.pdf]

Photos of all sampled canned cat food products and their codes in this study.

B1A	B1B	B1C	B1D
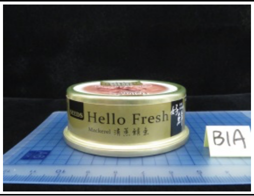	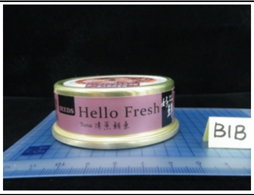	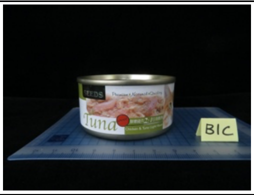	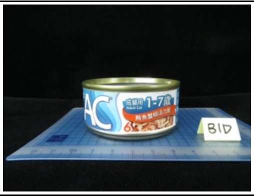
B2A	B2B	B2C	B2F
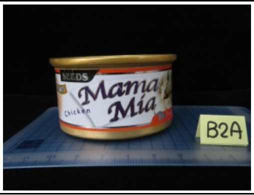	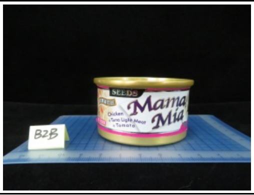	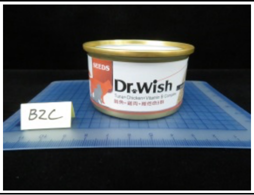	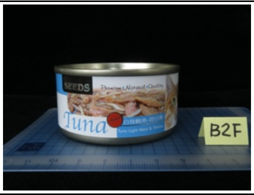
B2G	B2H	B3A	B3B
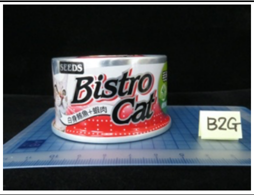	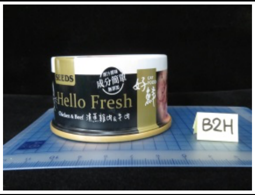	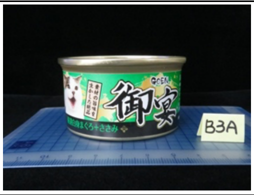	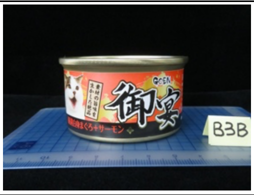
B3C	B3D	B3E	B3F
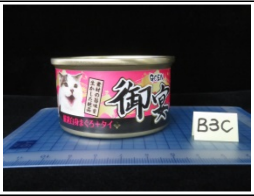	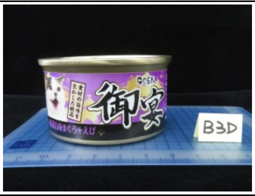	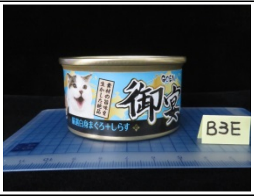	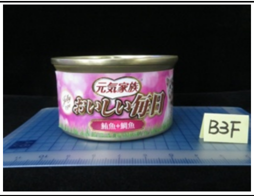
B3G	B3H	B3I	B3J
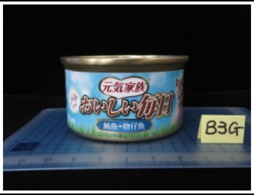	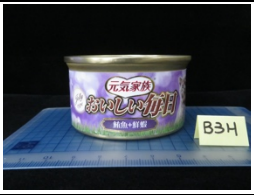	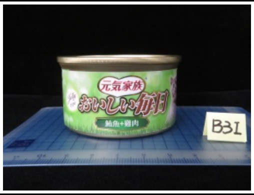	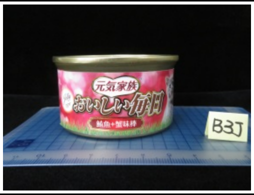
B3K	C1A	C1B	C1C
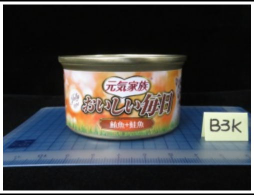	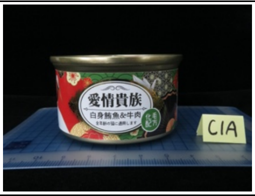	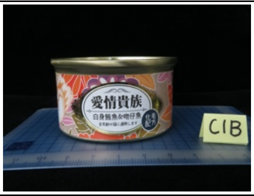	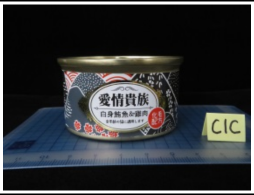
C1D	C1E	C1F	
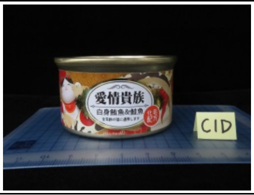	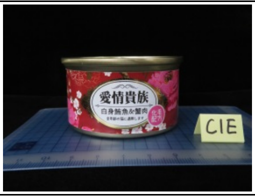	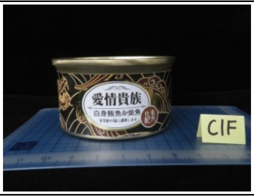	

C2A	C2B	C2C	C2D
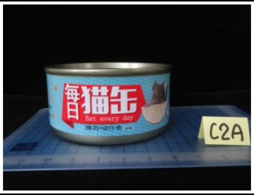	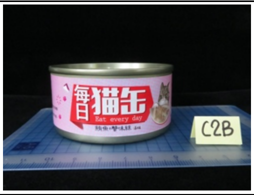	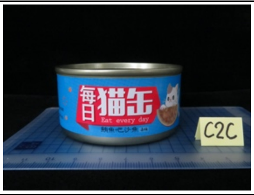	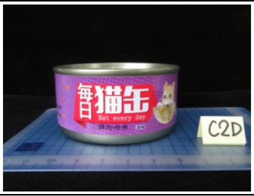
C3A	C3B	C3C	C3D
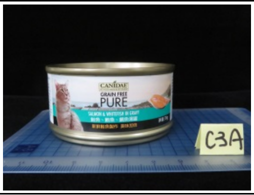	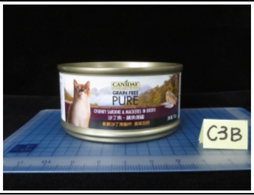	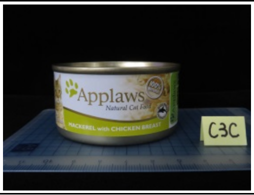	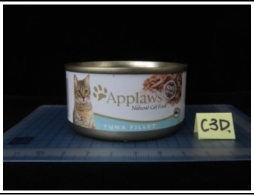
C3E	C4A	C4B	C4C
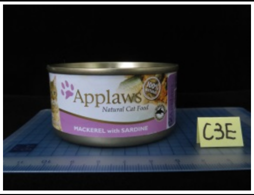	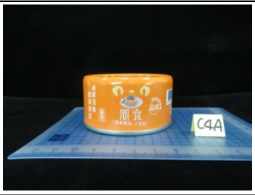	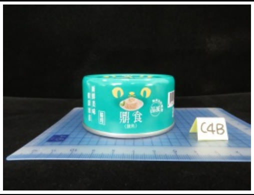	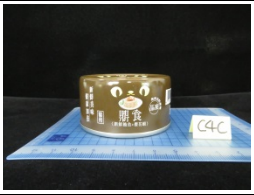
C4D	C5A	C5B	C5C
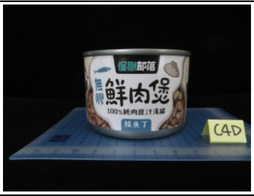	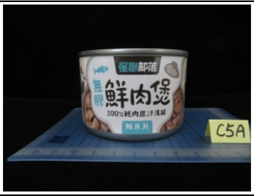	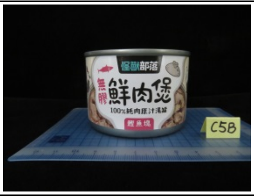	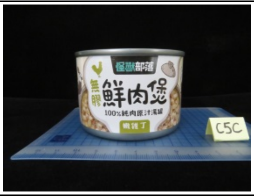
C5G	C5H	C5I	C6A
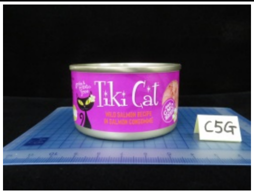	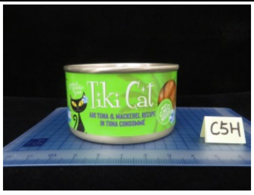	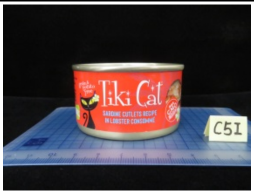	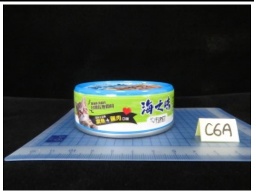
C6B	C6C	C6D	C6E
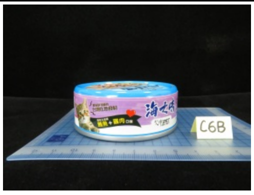	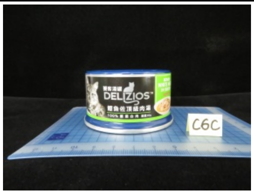	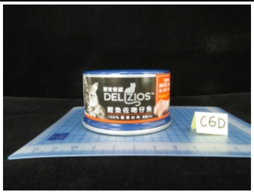	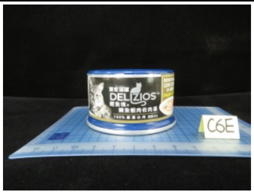
D3A	D3B	D3C	
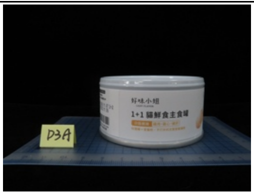	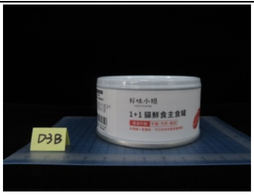	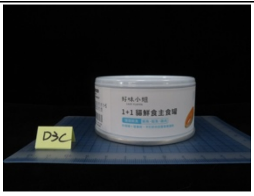	

D1A	D1B	D1C	D1D
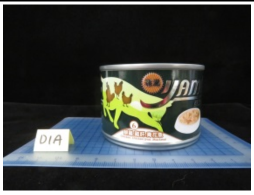	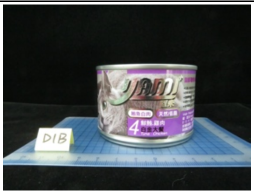	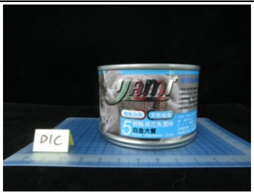	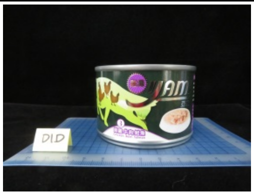
D2A	D2B	D2C	D2D
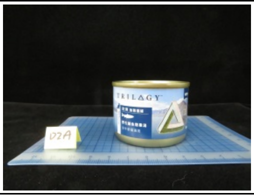	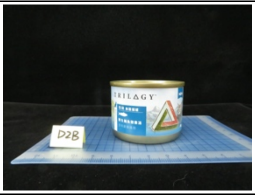	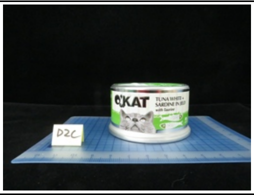	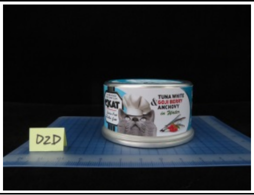
D2E	D2F	D2G	E1A
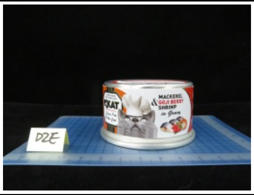	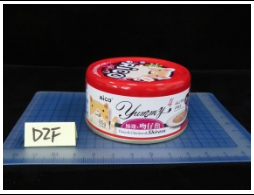	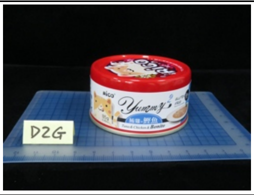	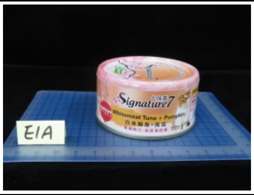
E1B	E1C	E1D	E1E
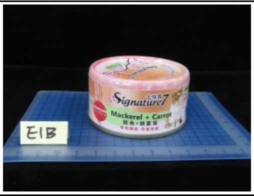	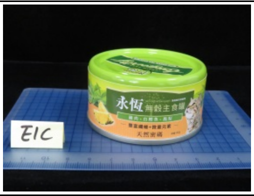	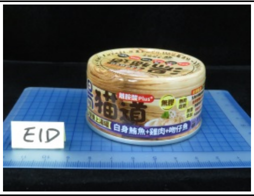	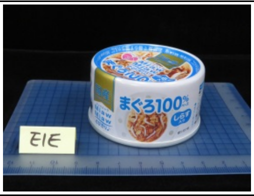
E1F	E2A	E2B	E2D
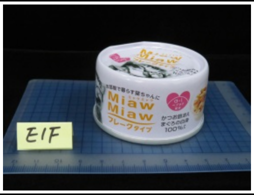	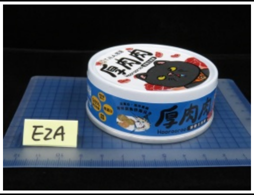	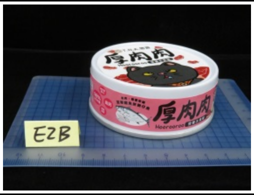	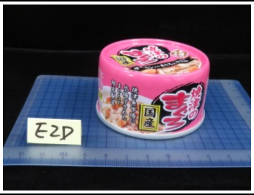
E2E	C5F	E3A	E3B
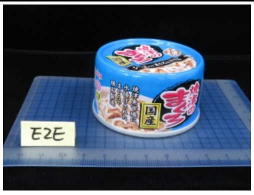	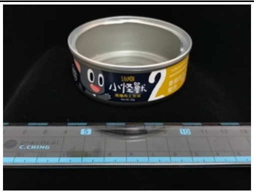	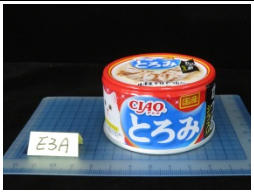	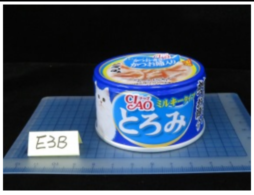
E3C	E3D	E3E	E4A
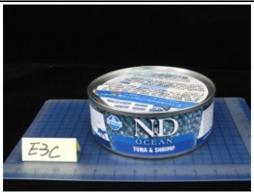	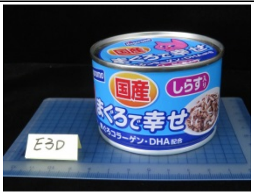	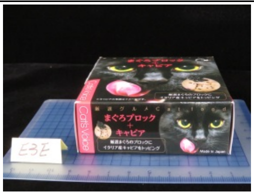	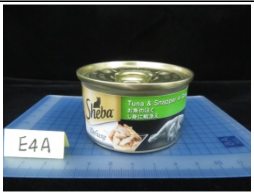

E4B	E4C	E4D	E4E
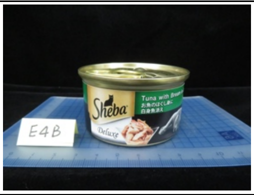	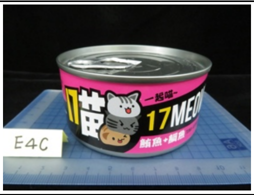	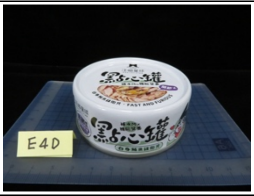	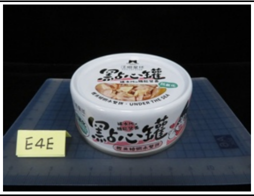
F2A	F2B	F2C	F2D
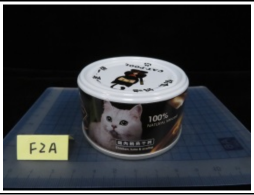	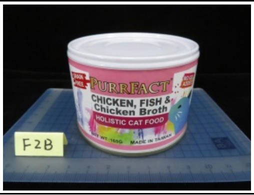	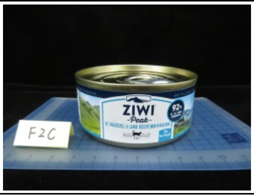	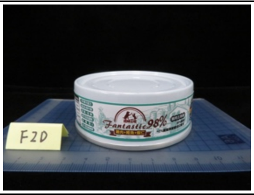
F2E	F2F	F3A	F3C
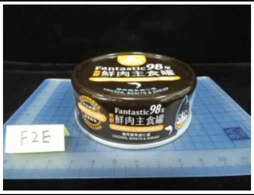	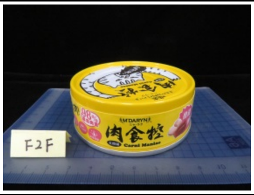	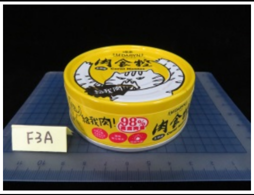	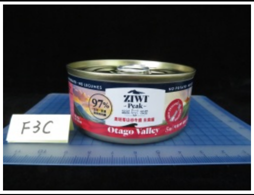
F3D	F1E	F4A	F4B
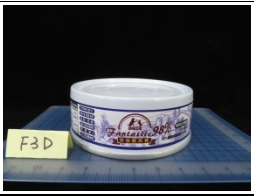	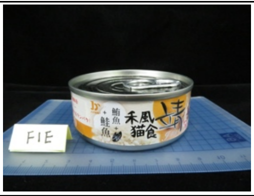	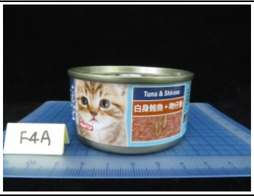	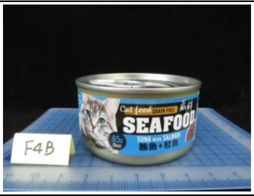
F4C	F4D	F5A	F5B
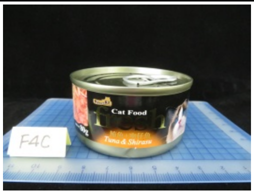	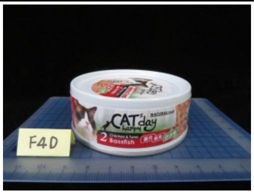	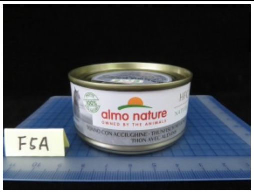	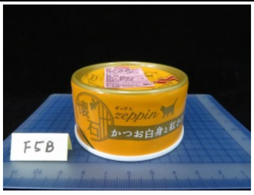
F5C	F5D	F5E	F6A
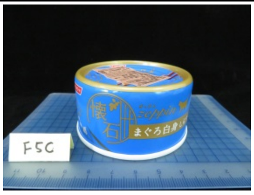	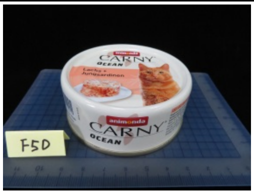	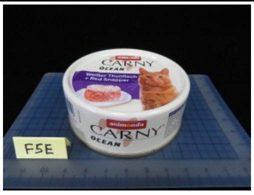	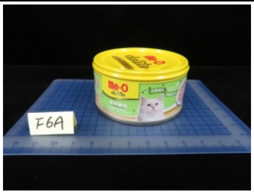
F6B	G1A	I1A	I1B
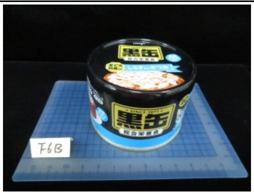	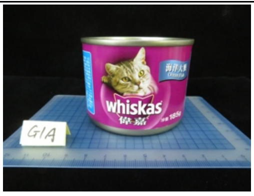	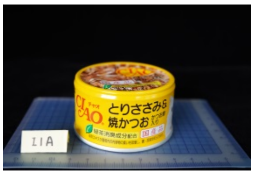	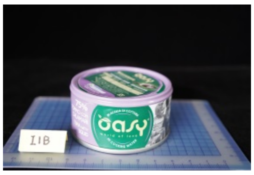

I1C	I2A	I2B	I2C
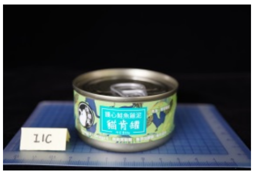	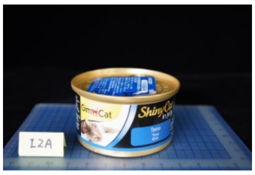	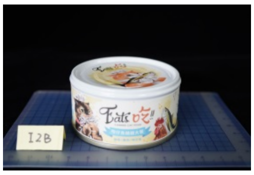	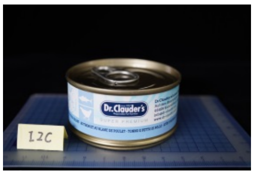
I2D	J1A	J1B	J1C
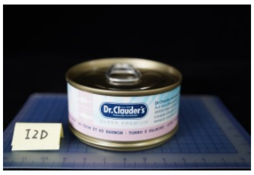	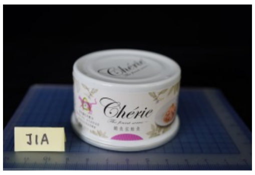	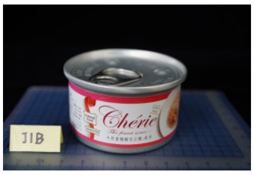	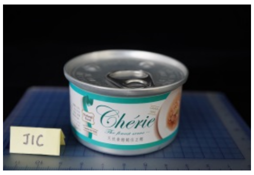
J1D	J2A	J2B	J2C
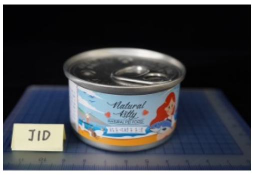	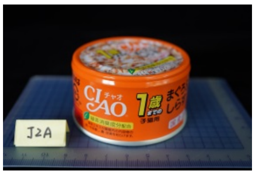	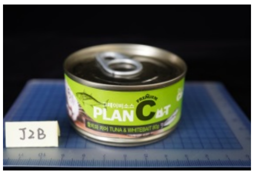	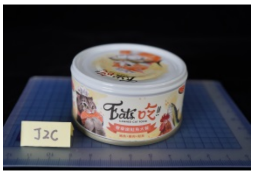
I3A	I3B	I3C	I3D
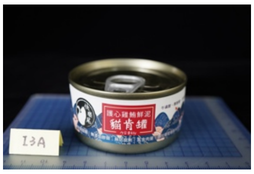	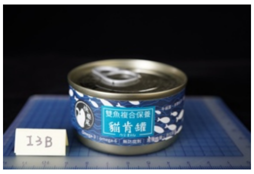	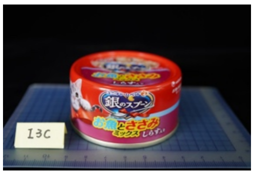	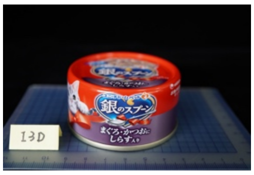
L1A	L1B	L1C	L1D
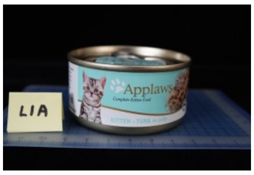	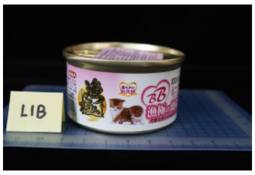	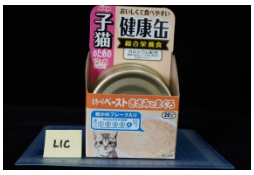	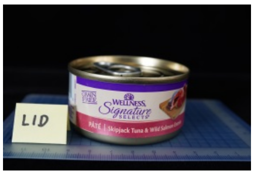
L2A	L2B	L2C	L2D
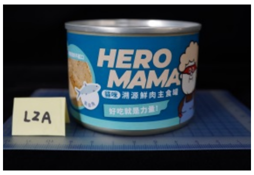	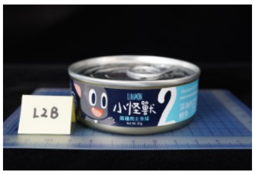	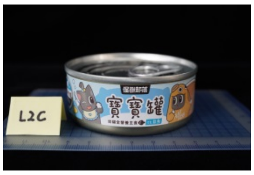	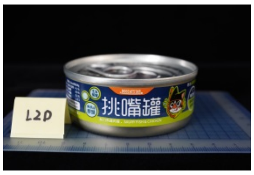
L3A	L3B	L3C	E2C
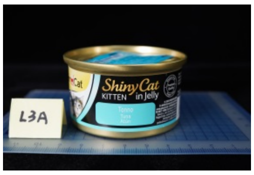	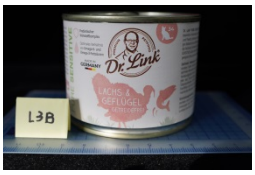	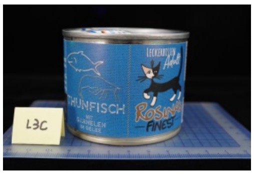	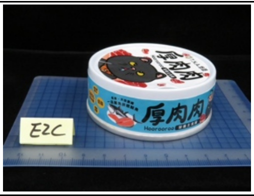
