# Supplementary material for: DNA mini-barcoding reveals the mislabeling rate of canned cat food in Taiwan
Source: PeerJ. 2024 Feb 21;12:e16833. doi: 10.7717/peerj.16833 (PMC10893872; doi:10.7717/peerj.16833)
Supplement: Supplemental Information 3 [file peerj-12-16833-s003.pdf]

>B1A
ATGGAGCTTTAGACACTAAGACAGTATCATGTTAAACACCCCACAAATAACGGCCTAAA
CTTAATGACCTCCTGTCCTAATGTCTTCG-------------------------------
>B1B
ATGGAGCTTTAGACACCAAGGCATATCATGTTAAACACCCCTAAATAAAGGACCAAACC
AAATGAATTATGCCCCCATGTCTTTG----------------------------------
>B1C1
ATGGAGCTTTAGACACCAAGGCATATCATGTTAAACACCCCTAAATAAAGGACCAAACC
AAATGAATTATGCCCCCATGTCTTTG----------------------------------
>B1C2
GTGGAACTTTAAAATCACGACCACCTTACAACCTTACACAGCCCCACTGGGTCCACCCA
CACATAAACCCCTGGTCGACATTTTTCG--------------------------------
>B1D1
ATGGAGCTTTAGACACTAAGGCATATCATGTTAAACACCCCTGAACAAAGGATTAAACC
AAATGAATAATGCCCCCATGTCTTTG----------------------------------
>B2A
GTGGAACTTTAAAATCACGACCACCTTACAACCTTACACAGCCCCACTGGGTCCACCCA
CACATAAACCCCTGGTCGACATTTTTCG--------------------------------
>B2B1
GTGGAACTTTAAAATCACGACCACCTTACAACCTTACACAGCCCCACTGGGTCCACCCA
CACATAAACCCCTGGTCGACATTTTTCG--------------------------------
>B2B2
ATGGAGCTTTAGACACTAAGGCATATCATGTTAAACACCCCTGAACAAAGGATTAAACC
AAATGAATAATGCCCCCATGTCTTTG----------------------------------
>B2F1
ATGGAGCTTTAGACACCAAGGCATATCATGTTAAACACCCCTAAATAAAGGACCAAACC
AAATGAATTATGCCCCCATGTCTTTG----------------------------------
>B2F2
ATGGAGCTTTAGACACTAGCCAAGTATGAATAAGCGGCCGCATTAAATGGCCCCAAGCA
CCCGTAGCCTTCTGGTAATGTGTCTTAT--------------------------------
>B2G1
ATGGAGCTTTAGACACCAAGGCATATCATGTTAAACACCCCTAAATAAAGGACCAAACC
AAATGAATTATGCCCCCATGTCTTTG----------------------------------
>B2H1
GTGGAACTTTAAAATCACGACCACCTTACAACCTTACACAGCCCCACTGGGTCCACCCA
CACATAAACCCCTGGTCGACATTTTTC---------------------------------
>B2H2
TTCATGGAGCTTTAACTAACCAACCCAAAGAGAATAGATTTAACCATTAAGGAATAACA

ACAATCTCCATGAGTTGGTAGTTTCG---------------------------------------
>B3A1
ATGGAGCTTTAGACACCAAGGCATATCATGTTAAACACCCCTAAATAAAGGACCAAACC
AAATGAATTATGCCCCCATGTCTTTG---------------------------------------
>B3A2
GTGGAACTTTAAAATCACGACCACCTTACAACCTTACACAGCCCCACTGGGTCCACCCA
CACATAAACCCCTGGTCGACATTTTTCG-------------------------------------
>B3B2
ATGGAGCTTTAGACACCAGGCAGATCACGTCAAGTAACCTTGAATTAACAAGTAAAAAC
GCAGTGACCCCTAGCCCATATGTCTTTG-------------------------------------
>B3B1
ATGGAGCTTTAGACACCAAGGCATATCATGTTAAACACCCCTAAATAAAGGACCAAACC
AAATGAATTATGCCCCCATGTCTTTG---------------------------------------
>B3C1
ATGGAGCTTTAGACACCAAGGCATATCATGTTAAACACCCCTAAATAAAGGACCAAACC
AAATGAATTATGCCCCCATGTCTTTG---------------------------------------
>B3C2
GTGGAGCTTTAGACGCTAAGGCAGACCGAGCCAAATTTGCCCGAGGACAATTAGCAAA
ACTAAATTCGAACCCTGCCCGCATGTCTTTG-----------------------------------
>B3D1
ATGGAGCTTTAGACACCAAGGCATATCATGTTAAACACCCCTAAATAAAGGACCAAACC
AAATGAATTATGCCCCCATGTCTTTG---------------------------------------
>B3D2
ATAAAGCTTTACAATTATTTAATTAAATTATAAATTGTTGGTTTAACTTGATTTAATTAATAT
TTGTTTC--
>B3E1
ATGGAGCTTTAGACACCAAGGCATATCATGTTAAACACCCCTAAATAAAGGACCAAACC
AAATGAATTATGCCCCCATGTCTTTG---------------------------------------
>B3E2
ATGGAGCTTTAGACACTAGCCAACTGTGAATAAGCGACTGAACTGAGCAAGTCCTAAAT
ACCCGCAGCCTTATGGTAATGTAGTCTTAG------------------------------------
>B3F1
ATGGAGCTTTAGACACTAAGGCATATCATGTTAAACACCCCTGAACAAAGGATTAAACC
AAATGAATAATGCCCCCATGTCTTTG---------------------------------------
>B3F2
GTGGAGCTTTAGACGCTAAGGCAGACCGAGCCAAATTTGCCCGAGGACAATTAGCAAA
ACTAAATTCGAACCCTGCCCGCATGTCTTTG-----------------------------------
>B3G2

ATGGAGCTTTAGACACTAGCCAATCTGTGAATAAGCGACTGAACTGAGCAAGTCCTAAA
TACCCGCAGCCTTATGGTAATGTAGTCTTAG----------------------------------
>B3I1
ATGGAGCTTTAGACACTAAGGCATATCATGTTAAACACCCCTGAACAAAGGATTAAACC
AAATGAATAATGCCCCCATGTCTTTG-------------------------------------
>B3H1
ATGGAGCTTTAGACACTAAGGCATATCATGTTAAACACCCCTGAACAAAGGATTAAACC
AAATGAATAATGCCCCCATGTCTTTG-------------------------------------
>B3J1
ATGGAGCTTTAGACACTAAGGCATATCATGTTAAACACCCCTGAACAAAGGATTAAACC
AAATGAATAATGCCCCCATGTCTTTG-------------------------------------
>B3I2
GTGGAACTTTAAAATCACGACCACCTTACAACCTTACACAGCCCCACTGGGTCCACCCA
CACATAAACCCCGTGGTCGACATTTTCG-----------------------------------
>B3J2
ATGGAGCTTTAGACACTAAGGCATATCATGTTAAACACCCCTGAACAAAGGATTAAACC
AAATGAATAATGCCCCCATGTCTTTG-------------------------------------
>B3K1
ATGGAGCTTTAGACACTAAGGCATATCATGTTAAACACCCCTGAACAAAGGATTAAACC
AAATGAATAATGCCCCCATGTCTTTG-------------------------------------
>C1A1
ATGGAGCTTTAGACACCAAGGCATATCATGTTAAACACCCCTAAATAAAGGACCAAACC
AAATGAACTATGCCCCCATGTCTTTG-------------------------------------
>C1B1
ATGGAGCTTTAGACACCAAGGCATATCATGTTAAACACCCCTAAATAAAGGACCAAACC
AAATGAATTATGCCCCCATGTCTTTG-------------------------------------
>C1D1
ATGGAGCTTTAGACACCAAGGCATATCATGTTAAACACCCCCAAATAAAGGACCAAACC
AAATGAATTATGCCCCCATGTCTTTG-------------------------------------
>C1C2
GTGGAACTTTAAAATCACGACCACCTTACAACCTTACACAGCCCCACTGGGTCCACCCA
CACATAAACCCCTGGTCGACATTTTCG------------------------------------
>C1E1
ATGGAGCTTTAGACACCAAGGCATATCATGTTAAACCACCCCTAAATAAAGGACCAAAC
CAAATGAATTATGCCCCCATGTCTTTG------------------------------------
>C1E2
ATGGAGCTTTAGACACCAAGGCATATCATGTTAAACACCCCTAAATAAAGGACCAAACC
AAATGAATTATGCCCCCATGTCTTTG-------------------------------------

>C1F1

ATGGAGCTTTAGACACCAAGGCATATCATGTTAAACACCCCTAAATAAAGGACCAAACC

AAATGAATTATGCCCCCATGT~~~~~~CTTTG---------------------------------

>C2A1

GTGGAACTTTAAAATCACGACCACCTTACAACCTTACACAGCCCCACTGGGTCCACCCA

CACATAAACCCCTGGTCGACATTTTTCG-------------------------------------

>C2A2

ATGGAGCTTTAGACACCAAGACAGATCATGTTAATGACCCTAAATAAAGGAACAAACCA

AATGGAATCTGTCCTAATGTCTTTG~---------------------------------------

>C2B1

ATGGAGCTTTAGACACCAAGGCATATCATGTCAAACACCCCTAAACAAAGGACTAAACC

AAATGAATCATAGCCCCCATGTCTTTG--------------------------------------

>C2C1

ATGGAGCTTTAGACACCAAGGCATATCATGTCAAACACCCCTAAACAAAGGACTAAACC

AAATGAATCATGACCCCCATGTCTTTG--------------------------------------

>C3A3

TATGGAGCTTTAGACACCAAGGCATATCATGTTAAACACCCCTAAATAAAGGACCAAAC

CAAATGAATTATGCCCCCATGTCTTTG--------------------------------------

>C2C2

TTGGAGCTTAAGATACAAGATCAACTATGTCAAGAACCCTAAAAGTTAAACTAAATAGC

AACTGATCCTTATCTTCG---

>C3A1

ATGGAGCTTTAGACACCCGGCAGATCACGTAAATAACCTTGAATTAACAAGTAAAAACG

CAGTGACCCCTAGCCCATATGTCTTTG--------------------------------------

>C3B1

ATGGAGCTTTAGACGCCCACCAACCGCGAAGAGCACCCCGACGACGGGCCACCAAATA

ACGCGACCATGGTATAAACGTCTTCG---------------------------------------

>C3C1

ATGGAGCTTTAAGACACTAAGCCATATCAAGTTAAATACCCCCTAACAAGGGGCCAAAC

TTATTGAAATCATTGGCCGTATGTCTTCG------------------------------------

>C3C2

GTGGAACTTTAAAATCACGACCACCTTACAACCTTACACAGCCCCACTGGGTCCACCCA

CACATAAACCCCTGGTCGACATTTTTCG-------------------------------------

>C3A2

ATGGAGCTTAAGGCGCCAGAACAGCTCACGTCAAACACCCCCGCATAAAGGGAATAAA

CCAAGTGGACCCTGCTCTAGTGTCTTTG-------------------------------------

>C3D

ATGGAGCTTTAGACACCAAGGCATATCATGTTAAACACCCCTAAACAAAGGGCTAAACC

AAATGAATCATGCCCCCATGTCTTTG---
>C3E1
ATGGAGCTTTAAGACATCTAAGCCATATCAAGTTAAATACCCCCTAACAAGGGGCCAAA
CTTATTGAAATCATTGGCCGTATGTCTTCG-------------------------------------
>C3E2
ATGGAGCTTTAAGACACTAAGCCATATCAAGTTAAATACCCCCTAACAAGGGGCCAAAC
TTATTGAAATCATTGGCCGTATGTCTTCG--------------------------------------
>C4A1
ATGGAGCTTTAGACACCAAGGCATATCATGTCAAACACCCCTAAACAAAGGACTAAACC
AAATGAATCATGCCCCCATGTCTTTG---
>C4A2
ATGGAGCTTTAGACACTAGCCAAGTATGAATAAGCGGCCGCATTAAATGGCCCCAAGCA
CCCGTAGCCTTCTGGTAATGTGTCTTAG---------------------------------------
>C2D1
GTGGAACTTTAAAATCACGACCACCTTACAACCTTACACAGCCCCACTGGGTCCACCCA
CACATAAACCCCTGGTCGACATTTTTCG---------------------------------------
>C4C1
ATGGAGCTTTAGACACCAAGGCATATCATGTCAAACACCCCTAAACAAAGGACTAAACC
AAATGAATCATGCCCCCATGTCTTTG---
>C4C2
CTATGGAGCTTTAGACACCAAGGCATATCATGTCAAACACCCCTAAACAAAGGACTAAA
CCAAATGAATCATGCCCCCATGTCTTTG---------------------------------------
>C4D
ATGGAGCTTTAAGACACTAAGCCATATCAAGTTAAATACCCCCTAACAAGGGGCCAAAC
TTATTGAAATCATTGGCCGTATGTCTTCG--------------------------------------
>C5A
ATGGAGCTTTAGACACCAAGGCATATCATGTTAAACACCCCTAAATAAAGGACCAAACC
AAATGAATTATGCCCCCATGTCTTTG---
>C5C
GTGGAACTTTAAAATCACGACCACCTTACAACCTTACACAGCCCCACTGGGTCCACCCA
CACATAAACCCCTGGTCGACATTTTTCG---------------------------------------
>C5B
ATGGAGCTTTAGACACCAAGGCATATCATGTTAAACACCCCTAAATAAAGGACCAAACC
AAATGAATTATGCCCCCATGTCTTTG---
>CC5G
TATGGAGCTTTAGACACCCGGCAGATCACGTAAAAAACCTTGAATTAACAAGTAAAAAC
GCAGTGACCCCTAGCCCATATGTCTTTG---------------------------------------
>C6A1

ATGGAGCTTTAGACACTAGGGCAGATCACGTTAAACAACTCCCAATAAAGAACAAAAC
TGAATGAGTCCTGCTTAAATGTCTTCG--------------------------------------
>C6A2
GTGGAACTTTAAAATCACGACCACCTTACAACCTTACACAGCCCCACTGGGTCCACCCA
CACATAAACCCCTGGTCGACATTTTTCG-------------------------------------
>C5I
GGAGCTTTAGACGCCCACCAACCTGCGAAGAGCACCCCGACGACGGGCCACCAAATAA
CGCGACCATGGTATAAACGTCTTCG---
>C6A3
TATGGAGCTTTAGACACTAGCCAAGTATGAATAAGCGGCCGCATTAAATGGCCCCAAGC
ACCCGTAGCCTTCTGGTAATGTGTCTTAG-------------------------------------
>C6A6
ATGGAGCTTTAGACACCAGCTAACTGTGAACTAGCGAATGAATAATAAATCTTCCCATAC
ACCCACAGACCCTGGTAATGTGTCTTAG~~~~~--------------------------------
>C6D2
ATGGAGCTTTAGACACCAGCTAACTGTGAAATAGGAATGAATAATAAATCTTCCCATACA
CCCACAGACCCTGGTAATGTGTCTTAG--------------------------------------
>C6E2
TATGGAGCTTTAGACACCAGAATAGCACATGTTAAGACACCCTGAATAAAGAACTGAAC
TTAATGGCCCCCCTATTCCAATGTCTTCG------------------------------------
>D3A
GTGGAACTTTAAAATCACGACCACCTTACAACCTTACACAGCCCCACTGGGTCCACCCA
CACATAAACCCCTGGTCGACATTTTTCG-------------------------------------
>D3B1
ATGGAGCTTTAACTAACCAACCCAAAGAGAATAGATTTAACCATTAAGGAATAACAACA
ATCTCCATGAGTTGGTAGTTTCG--
>D3B2
GTGGAACTTTAAAATCACGACCACCTTACAACCTTACACAGCCCCACTGGGTCCACCCA
CACATAAACCCCTGGTCGACATTTTTCG-------------------------------------
>D3C2
GTGGAACTTTAAAATCACGACCACCTTACAACCTTACACAGCCCCACTGGGTCCACCCA
CACATAAACCCCTGGTCGACATTTTTCG~~~~~~~------------------------------
>D3C3
GTGGAACTTTAAAATCACGACCACCTTACAACCTTACACAGCCCCACTGGGTCCACCCA
CACATAAACCCCTGGTCGACATTTTTCG-------------------------------------
>D38_16F
GTGGAACTTTAAAATCACGACCACCTTACAACCTTACACAGCCCCACTGGGTCCACCCA
CACATAAACCCCTGGTCGACATTTTTCG~------------------------------------

>D1B1
ATGGAGCTTTAGACACCAAGGCATATCATGTTAAACACCCCTAAACAAAGGGCTAAACC
AAATGAATCATGCCCCCATGTCTTTG---
>D1B2
GTGGAACTTTAAAATCACGACCACCTTACAACCTTACACAGCCCCACTGGGTCCACCCA
CACATAAACCCCTGGTCGACATTTTTCG---------------------------------------
>D1C1
ATGGAGCTTTAGACACCAAGGCATATCATGTTAAACACCCCTAAACAAAGGGCTAAACC
AAATGAATCATGCCCCCATGTCTTTG---
>D1C2
ATGGAGCTTTAAGACATAAGCCATATCAAGTTAAATACCCCCTAACAAGGGGCCAAACT
TATTGAAATCATTGGCCGTATGTCTTCG~--------------------------------------
>D1D1
GTGGAACTTTAAAATCACGACCACCTTACAACCTTACACAGCCCCACTGGGTCCACCCA
CACATAAACCCCTGGTCGACATTTTTCG---------------------------------------
>D1D2
ATGGAGCTTTAGACACCCGGCAGATCACGTAAAAAACCTTGAATTAACAAGTAAAAAC
GCAGTGACCCCTAGCCCATATGTCTTTG~---------------------------------------
>D1D3
ATGGAGCTTTAACTAACCAACCCAAAGAGAATAAATTTAACCATTAAGGAGTAACAACA
ATCTCCATGAGTTGGTAGTTTCG--
>D2A
ATGGAGCTTTAAGACACTAAGCCATATCAAGTTAAATACCCCCTAACAAGGGGCCAAAC
TTATTGAAATCATTGGCCGTATGTCTTCG--------------------------------------
>D2B
ATGGAGCTTTAGACACCAAGGCATATCATGTTAAACACCCCTAAATAAAGGACCAAACC
AAATGAATTATGCCCCCATGTCTTTG---
>D3C1
TATGGAGCTTTCAGACGCCAGAACAGACCACGTTAAGCACTCCTGAAATAAAGGATAA
AACTGATTGACCCCTGTCCTAATGTCTTTG--------------------------------------
>D2C1
ATGGAGCTTTAGACACCAAGGCATATCATGTTAAACACCCCTAAATAAAGGACCAAACC
AAATGAATTATGCCCCCATGTCTTTG---
>D2C2
ATGGAGCTTTAGACGCCCACCAACCGCGAAGAGCACCCCGACGACGGGCCACCAAATA
ACGCGACCATGGTATAAACGTCTTCG--
>D2D2
TATGGAGCTTTAGACACCAAGGCATATCATGTTAAACACCCCTAAATAAAGGACCAAAC

CAAATGAATTATGCCCCCATGTCTTTG---
>D2E2
ATAAAGCTTTACAATTATTTAATTAAATTATAAATTGTTGGTTTAACTTGATTTAATTAATAT
TTGTTTC---
>D2F2
GTGGAACTTTAAAATCACGACCACCTTACAACCTTACACAGCCCCACTGGGTCCACCCA
CACATAAACCCCTGGTCGACATTTTTCG~--
>D2F3
ATGGAGCTTTAGACACCAGCTAACTGTGAACTAGCGAATGAATAATAAATCTTCCCATAC
ACCCACAGACCCTGGTAATGTGTCTTAG---
>D2G2
GTGGAACTTTAAAATCACGACCACCTTACAACCTTACACAGCCCCACTGGGTCCACCCA
CACATAAACCCCTGGTCGACATTTTTCG--
>D2G3
ATGGAGCTTTAGACACCAAGGCATATCATGTCAAACACCCCTAAACAAAGGACTAAACC
AAATGAATCATGCCCCCATGTCTTTG--
>E1C1
GTGGAACTTTAAAATCACGACCACCTTACAACCTTACACAGCCCCACTGGGTCCACCCA
CACATAAACCCCTGGTCGACATTTTTCG--
>E1D2
GTGGAACTTTAAAATCACGACCACCTTACAACCTTACACAGCCCCACTGGGTCCACCCA
CACATAAACCCCTGGTCGACATTTTTCG--
>E1E1
ATGGAGCTTTAGACACCAAGGCATATCATGTCAAACACCCCTAAACAAAGGACTAAACC
AAATGAATCATGCCCCCATGTCTTTG--
>E21_16F
ATGGAGCTTTAGACACTAGCCAAGTATGAATAAGCGGCCGCATTAAATGGCCCCAAGCA
CCCGTATCCTTCTGGTAATGTGTCTTAT--
>E1F1
ATGGAGCTTTAGACACCAAGGCATATCATGTCAAACACCCCTAAACAAAGGACTAAACC
AAATGAATCATGCCCCCATGTCTTTG--
>E2A
ATGGAGCTTTAGACACTAGGGCAGATCACGTTAAACAACTCCCAATAAAGAACAAAAC
TGAATGAGTCCTGCTTAAATGTCTTCG---
>E2C
AGGGAGCTTTAGACACCAAGGCATATCATGTCAAACACCCCTAAACAAGGGACTAAAC
CAAATGAATCATGCCCCCATGTCTTTG---
>E2D1

ATGGAGCTTTAGACACCAAGGCATATCATGTCAAACACCCCTAAACAAAGGACTAAACC
AAATGAATCATGCCCCCATGTCTTTG-------------------------------------
>E2D2
GTGGAACTTTAAAATCACGACCACCTTACAACCTTACACAGCCCCACTGGGTCCACCCA
CACATAAACCCCTGGTCGACATTTTTCG~------------------------------------
>E2D3
ATGGAGCTTTAGACACCAGGCAGATCACGTCAAAAACCTTGAATTAACAAGTAAAAAC
GCAGTGACCCCTAGCCCATATGTCTTTG-------------------------------------
>E2E1
ATGGAGCTTTAGACACCAAGGCATATCATGTCAAACACCCCTAAACAATAGGACTAAAC
CAAATGAATCATGCCCCCATGTCTTTG-------------------------------------
>E2E3
ATGGAGCTTTAGACACCAGCTAACTGTGAACTAGCGAATGAATAATAAATCTTCCCATAC
ACCCACAGACCCTGGTAATGTGTCTTAG------------------------------------
>C5F2
GTGGAACTTTAAAATCACGACCACCTTACAACCTTACACAGCCCCACTGGGTCCACCCA
CACATAAACCCCTGGTCGACATTTTTCG-------------------------------------
>E3A1
GTGGAACTTTAAAATCACGACCACCTTACAACCTTACACAGCCCCACTGGGTCCACCCA
CACATAAACCCCTGGTCGACATTTTTCG-------------------------------------
>E3A2
GTGGAACTTTAAAATCACGACCACCTTACAACCTTACACAGCCCCACTGGGTCCACCCA
CACATAAACCCCTGGTCGACATTTTTCG~------------------------------------
>E3B1
GTGGAACTTTAAAATCACGACCACCTTACAACCTTACACAGCCCCACTGGGTCCACCCA
CACATAAACCCCTGGTCGACATTTTTCG-------------------------------------
>E3B2
GTGGAACTTTAAAATCACGACCACCTTACAACCTTACACAGCCCCACTGGGTCCACCCA
CACATAAACCCCTGGTCGACATTTTTCG-------------------------------------
>E3B3
ATGGAGCTTTAGACACCAAGGCATATCATGTTAAACACCCCTAAACAAAGGACTAAACC
AAATGAATCATGCCCCCATGTCTTTG-------------------------------------
>E3C1
ATGGAGCTTTAGACACCAAGGCATATCATGTCAAACACCCCTAAACAAAGGACTAAACC
AAATGAATCATGCCCCCATGTCTTTG~------------------------------------
>E3D1
ATGGAGCTTTAGACACCAAGGCATATCATGTCAAACACCCCTAAACAAAGGACTAAACC
AAATGAATCATGCCCCCATGTCTTTG-------------------------------------

>E3D2
ATGGAGCTTTAGACACCAGCTAACTGTGAACTAGCGAATGAATAATAAATCTTCCCATGC
ACCCACAGACCCTGGTAATGTGTCTTAG~------------------------------------
>E3E1
ATGGAGCTTTAGACACCAAGGCATATCATGTCTAAACACCCCTAAACAAAGGACTAAAC
CAAATGAATCATGCCCCCATGTCTTTG--
>E4A1
ATGGAGCTTTAGACACCAAGGCATATCATGTTAAACACCCCTAAACAAAGGACTAAACC
AAATGAATCATGCCCCCATGTCTTTG---
>E4A2
GTGGAGCTTTAGACGCTAAGACAGATCTTATTAAATTTATCCTAAAATAACAGGATAAAA
ACTAAATTAGAACCCTGCCTGCATGTCTTTG----------------------------------
>E4B1
ATGGAGCTTTAGACACCAAGGCATATCATGTCTAAACACCCCTAAACAAAGGACTAAAC
CAAATGAATCATGCCCCCATGTCTTTG~--------------------------------------
>F6_16F
ATGGAGCTTTAGACACCAAGGCATATCATGTTAAACACCCCTAAATAAAGGACCAAACC
AAATGAATTATGCCCCCATGTCTTTG--
>E4D1
ATGGAGCTTTAGACACCAAGGCATATCATGTTAAACACCCCTAAATAAAGGACTAAACC
AAATGAATTATGCCCCCATGTCTTTG~---------------------------------------
>E4E1
ATGGAGCTTTAGACACCAAGGCATATCATGTTAAACACCCCTAAATAAAGGACTAAACC
AAATGAATTATGCCCCCATGTCTTTG--
>E4E2
ATGGAGCTTTAGACACCAAGGCATATCATGTTAAACACCCCTAAACAAAGGACTAAACC
AAATGAATCATGCCCCCATGTCTTTG--
>F1E1
ATGGAGCTTTAGACACCAAGGCATATCATGTTAAACACCCCTAAATAAAGGACCAAACC
AAATGAATTATGCCCCCATGTCTTTG--
>F4A1
ATGGAGCTTTAGACACCAAGGCATATCATGTTAAACACCCCTAAATAAAGGACCAAACC
AAATGAATTATGCCCCCATGTCTTTG--
>F4A2
ATGGAGCTTTAGACACCAGCCAACTGTGAAATAGCGATTAATACACCTTATTCCCAAACA
TCCGCAGCCTCTGGTAAAGTGTCTTAG--------------------------------------
>F4C1
ATGGAGCTTTAGACACCAAGGCATATCATGTCAAACACCCCTAAACAAAGGACTAAACC

AAATGAATCATGCCCCCATGTCTTTG---------------------------------------
>F4D1
GTGGAACTTTAAAATCACGACCACCTTACAACCTTACACAGCCCCACTGGGTCCACCCA
CACATAAACCCCTGGTCGACATTTTTCG-------------------------------------
>F4D2
ATGGAGCTTTAGACACCAAGGCATATCATGTCAAACACCCCTAAACAAAGGACTAAACC
AAATGAATCATGCCCCCATGTCTTTG---------------------------------------
>F4D3
TTGGAGCTTAAGATATAAGATCAACTATGTCAAGAACCCTAAAAGTTAAACTAAATAGCA
ACTGATCCTTATCTTCG---
>G10
ATGGAGCTTTAGACACCAAGGCATATCATGTTAAACACCCCTAAATAAAGGACCAAACC
AAATGAATTATGCCCCCATGTCTTTG---------------------------------------
>G11
ATGGAGCTTTAGACACCAAGGCAGAACATGTTAAGTACCCCTAAACAAGGGCCGAACC
GAATGAACCCTGCCCCAATGTCTTTG--
>F5A1
ATGGAGCTTTAGACACCAAGGCATATCATGTTAAACACCCCTAAACAAAGGGCTAAACC
AAATGAATCATGCCCCCATGTCTTT--
>F5A2
ATGGAGCTTTAGACGCCCACCAACCGCGAAGAGCACCCCGACGACGGGCCACCAAATA
ACGCGACCATGGTATAAACGTCTTCG--
>F5B1
ATGGAGCTTTAGACACCAAGGCATATCATGTTAAACACCCCTAAATAAAGGACCAAACC
AAATGAATTATGCCCCCATGTCTTTG---------------------------------------
>F5C1
ATGGAGCTTTAGACACCAAGGCATATCATGTCAAACACCCCTAAACAAAGGACTAAACC
AAATGAATCATGCCCCCATGTCTTTG---------------------------------------
>F5C2
ATGGAGCTTTAGACACTAGCCAACTGTGAATAAGCGACTGAACTGAGCAAGTCCTAAAT
ACCCGCAGCCTTATGGTAATGTAGTCTTAG~----------------------------------
>F5D1
ATGGAGCTTTAGACACCCGGCAGATCACGTAAAAAACCTTGAATTAACAAGTAAAAAC
GCAGTGACCCCTAGCCCATATGTCTTTG--------------------------------------
>F5D2
ATGGAGCTTTAGACACCAGCTAACTGTGAACTAGCGAATGAATAATAAATCTTCCCATAC
ACCCACAGACCCTGGTAATGTGTCTTAG------------------------------------
>F6A

ATGGAGCTTTAGACACCAAGGCATATCATGTTAAACACCCCTAAATAAAGGACCAAACC
AAATGAATTATGCCCCCATGTCTTTG--
>F6B1
TGGAGCTTTAGACACCAAGGCATATCATGTTAAACACCCCTAAATAAAGGACCAAACCA
AATGAATTATGCCCCCATGTCTTTG--
>F6B2
ATGGAGCTTTAGACACCAAGGCATATCATGTTAAACACCCCTAAATAAAGGACCAAACC
AAATGAATTATGCCCCCATGTCTTTG--
>F6B3
ATGGAGCTTTAGACACCAAGGCATATCATGTTAAACACCCCTAAATAAAGGACCAAACC
AAATGAATTATGCCCCCATGTCTTTGGT-------------------------------------
>G1A1
ATGGAGCTTTAGACACCAAGGCATATCATGTTAAACACCCCTAAATAAAGGACCAAACC
AAATGAATTATGCCCCCATGTCTTTG--
>G1A2
ATGGAGCTTTAGACACCAAGGCATATCATGTTAAACACTCCCTAAATAAAGGACCAAAC
CAAATGAATTATGCCCCCATGTCTTTG---------------------------------------
>I2B2
GTGGAACTTTAAAATCACGACCACCTTACAACCTTACACAGCCCCACTGGGTCCACCCA
CACATAAACCCCTGGTCGACATTTTTCG--------------------------------------
>I2B3
ATGGAGCTTTAGACACTAGCCAAGTATGAATAAGCGGCCGCATTAAATGGCCCCAAGCA
CCCGTAGCCTTCTGGTAATGTGTCTTAG--------------------------------------
>J1A1
ATGGAGCTTTAGACACCAAGGCATATCATGTTAAACACCCCTAAATAAAGGACCAAACC
AAATGAATTATGCCCCCATGTCTTTG--
>J1A2
ATGGAGCTTTAGACACCCGGCAGATCACGTAAATAACCTTGAATTAACAAGTAAAAACG
CAGTGACCCCTAGCCCAT~~~~~~~~~~~~~~~~~~~~~~~~~~~~~~~~~~~~~~ATGTCTTTG
>J1B1
ATGGAGCTTTAGACACCAAGGCATATCATGTTAAACACCCCTAAATAAAGGACCAAACC
AAATGAATTATGCCCCCATGTCTTTG--
>J1B2
ATGGAGCTTTAGACACCCGGCAGATCACGTAAAAAACCTTGAATTAACAAGTAAAAAC
GCAGTGACCCCTAGCCCATATGTCTTTG--------------------------------------
>J1B3
ATGGAGCTTTAGACACCCGGCAGATCACGTAAAAAACCTTGAATTAACAAGTAAAAAC
GCAGTGACCCCTAGCCCATATGTCTTTG--------------------------------------

>J1C1
ATGGAGCTTTAGACACCAAGGCATATCATGTTAAACACCCCTAAATAAAGGACCAAACC
AAATGAATTATGCCCCCATGTCTTTG-------------------------------------
>J1C2
ATGGAGCTTTAGACACCAAGGCATATCATGTCAAACACCCCTAAACAAAGGACCTAAAC
CAAATGAATCATGCCCCCATGTCTTTG-------------------------------------
>J1D1
ATGGAGCTTTAGACACCAAGGCATATCATGTTAAACACCCCTAAATAAAGGACCAAACC
AAATGAATTATGCCCCCATGTCTTTG-------------------------------------
>J1D2
ATGGAGCTTTAGACACTAGCCAAGTATGAATAAGCGGCCGCATTAAATGGCCCCAAGCA
CCCGTAGCCTTCTGGTAATGTGTCTTAG-----------------------------------
>J2A1
ATGGAGCTTTAGACACCAAGGCATATCATGTCAAACACCCCTGAACAAAGGACTAAACC
AAATGAATCATGCCCCCATGTCTTTG-------------------------------------
>J2A2
ATGGAGCTTTAGACACTAGCCAACTGTGAATAAGCGACTGAACTGAGCAAGTCCTAAAT
ACCCGCAGCCTTATGGTAATGTAGTCTTAG---------------------------------
>J2B1
ATGGAGCTTTAGACACCAAGGCATATCATGTTAAACACCCCTAAATAAAGGACCAAACC
AAATGAATTATGCCCCCATGTCTTTG-------------------------------------
>J2B2
ATGGAGCTTTAGACACTAGCCAAGTATGAATAAGCGGCCGCATTAAATGGCCCCAAGCA
CCCGTAGCCTTCTGGTAATGTGTCTTAG-----------------------------------
>J2C2
GTGGAACTTTAAAATCACGACCACCTTACAACCTTACACAGCCCCACTGGGTCCACCCA
CACATAAACCCCTGGTCGACATTTTTCG-----------------------------------
>I3B2
ATGGAGCTTTAGACACTAGGGCAGATCACGTTAAACAACTCCCAATAAAGAACAAAAC
TGAATGAGTCCTGCTTAAATGTCTTCG-------------------------------------
>I3C1
GTGGAACTTTAAAATCACGACCACCTTACAACCTTACACAGCCCCACTGGGTCCACCCA
CACATAAACCCCTGGTCGACATTTTTCG-----------------------------------
>I3C4
GTGGAACTTTAAAATCACGACCACCTTACAACCTTACACAGCCCCACTGGGTCCACCCA
CACATAAACCCCTGGTCGACATTTTTCG-----------------------------------
>I3D1
ATGGAGCTTTAGACACCAAGGCATATCATGTTAAACACCCCTAAATAAAGGACCAAACC

AAATGAATTATGCCCCCATGTCTTTG--------------------------------------
>I3D2
ATGGAGCTTTAGACACCAAGGCATATCATGTTAAACACCCCTAAATAAAGGACCAAACC
AAATGAATCATGCCCCCATGTCTTTG--------------------------------------
>I1A1
GTGGAACTTTAAAATCACGACCACCTTACAACCTTACACAGCCCCACTGGGTCCACCCA
CACATAAACCCCTGGTCGACATTTTTCG------------------------------------
>I1A2
ATGGAGCTTTAGACACCAAGGCATATCATGTTAAACACCCCTAAATAAAGGACCAAACC
AAATGAATTATGCCCCCATGTCTTTG--------------------------------------
>I1B1
ATGGAGCTTTAGACACCAAGGCATATCATGTTAAACACCCCTAAATAAAGGACCAAACC
AAATGAATTATGCCCCCATGTCTTTG~-------------------------------------
>I2B1
ATGGAGCTTTAGACACCAAGGCATATCATGTTAAACACCCCTAAATAAAGGACCAAACC
AAATGAATTATGCCCCCATGTCTTTG--------------------------------------
>I6_M13F(-20)
GTGGAACTTTAAAATCACGACCACCTTACAACCTTACACAGCCCCACTGGGTCCACCCA
CACATAAACCCCTGGTCGACATTTTTCG------------------------------------
>I2D2
ATGGAGCTTTAGACACCCGGCAGATCACGTAAAAAACCTTGAATTAACAAGTAAAAAC
GCAGTGACCCCTAGCCCATATGTCTTTG~-----------------------------------
>C1D2
ATGGAGCTTTAGACACCAGGCAGATCACGTCAAAAACCTTGAATTAACAAGTAAAAAC
GCAGTGACCCCTAGCCCATATGTCTTTG------------------------------------
>E4B2
GTGGAGCTTTAGGTACTAAGGTTGTCCCACTAAACATATTCAAATAAAAGTATTCAAGAA
GGGCATCAATCCGATTATCTTTG---
>F4C2
ATGGAGCTTTAGACACTAGCCAAGTATGAATAAGCGGCCGCATTAAATGGCCCCAAGCA
CCCGTAGCTTCTGGTAATGTGTCTTAG-------------------------------------
>C1F2
ATGGAGCTTTAGACACCAAGGCATATCATGTTAAACACCCCTAAATAAAGGGCTAAACC
AAATGAATCATGCCCCCATGTCTTTG--------------------------------------
>E2B2
ATGGAGCTTTAGACACTAGCCAAGTATGAATAAGCGGCCGCATTAAATGGCCCCAAGCA
CCCGTAGCCTTCTGGTAATGTGTCTTAG------------------------------------
>I2D1

AGGGAGCTTTAGACACCAAGGCATATCATGTTAAACACCCCTAAATAAAGGACCAAACC
AAATGAATTATGCCCCCATGTCTTTG--
>F5B2
ATGGAGCTTTAGACACCAGGCAGATCACGTCAAAAACCTTGAATTAACAAGTAAAAAC
GCAGTGACCCCCTAGCCCATATGTCTTTG--
>E3E2
ATGGAGCTTAAAACACAAGATCAACTATGCTATCAAGCCAACCACCCACGGAAATAACA
GCTAAAAGCATAATAGTACCCTGATCCTAATGTTTTCG-----------------------------
>E4D2
ATGGAGCTTTAGACACCAAGGCATATCATGTTAAACACCCCTAAACAAAGGACTAAACC
AAATGAATCATGCCCCCATGTCTTTG--
>L1A
ATGGAGCTTTAGACACCAAGGCATATCATGTTAAACACCCCTAAACAAAGGGCTAAACC
AAATGAATCATGCCCCCATGTCTTTG--
